# DNA bipedal motor walking dynamics: an experimental and theoretical study of the dependency on step size

**DOI:** 10.1093/nar/gkx1282

**Published:** 2017-12-27

**Authors:** Dinesh C Khara, John S Schreck, Toma E Tomov, Yaron Berger, Thomas E Ouldridge, Jonathan P K Doye, Eyal Nir

**Affiliations:** 1 Ben-Gurion University of the Negev, Beer-Sheva and the Ilse Katz Institute for Nanoscale Science and Technology, P.O. Box 653, Beer-Sheva, 8410501, Israel; 2 Department of Chemical Engineering, Columbia University, 500 W 120th Street, New York, NY 10027, USA; 3 Department of Bioengineering, Imperial College London, South Kensington Campus, London SW7 2AZ, UK; 4 Physical and Theoretical Chemistry Laboratory, Department of Chemistry, University of Oxford, South Parks Road, Oxford OX1 3QZ, UK

## Abstract

We present a detailed coarse-grained computer simulation and single molecule fluorescence study of the walking dynamics and mechanism of a DNA bipedal motor striding on a DNA origami. In particular, we study the dependency of the walking efficiency and stepping kinetics on step size. The simulations accurately capture and explain three different experimental observations. These include a description of the maximum possible step size, a decrease in the walking efficiency over short distances and a dependency of the efficiency on the walking direction with respect to the origami track. The former two observations were not expected and are non-trivial. Based on this study, we suggest three design modifications to improve future DNA walkers. Our study demonstrates the ability of the oxDNA model to resolve the dynamics of complex DNA machines, and its usefulness as an engineering tool for the design of DNA machines that operate in the three spatial dimensions.

## INTRODUCTION

Biological macromolecular machines, which play major roles in many biological processes, often operate with remarkably high chemical yield and speed. For example, the bipedal kinesin motor can perform hundreds of steps per second before dissociating from the microtubule track (1,2). Recent advances in structural DNA nanotechnology (3), including DNA origami technology (4–8), and our understanding of DNA dynamics (9) offers a unique path toward the realization of synthetic molecular machines (10–12). Impressive tweezers (13), molecular assembly lines (14,15), gears (16), crank sliders (17), pullers (18), rotors (19), walkers (20–37) and robots (38) made from DNA have been demonstrated. However, the number of operations (e.g. the number of steps for a walker) demonstrated thus far is significantly lower than that of biological machines. To advance the field of synthetic DNA molecular machines, therefore, major efforts need to be focused on achieving machines with significantly improved performance. A prerequisite for the rational design of such devices is both a good understanding of the machine operation mechanisms and dynamics, and also the reasons for machine failure.

To meet this challenge, we have previously developed (based on an earlier design (22)) a DNA-based bipedal walker model system that strides on an origami track (34–36). The walker legs are connected to the track footholds via DNA strands called ‘fuels’. Using a microfluidics device, which allows the introduction and removal of fuel and ‘antifuel’ strands from the motor solution, we demonstrated 32 steps with 44% total operational yield (35). However, this is still noticeably less than the hundreds of steps achieved by kinesin (1,2). We studied the dependency of the motor performance on fuel concentrations and found that the main factor that limits the yield was a failure to sometimes complete the fuel-mediated attachment of the walker leg to the foothold, a reaction we call ‘leg placing’ (34,35). One of the potential ways to optimize the yield of this reaction, and hence walker performance, is through the size of the walker step. However, to the best of our knowledge, the experimental dependency of a DNA walker’s performance on step size has not yet been reported for any DNA walker.

The well established structural, thermodynamic and dynamical properties of single- and double-stranded DNA (39), and structural properties of DNA origami (40–42) although very useful, may not provide sufficient information to enable the design of complex DNA machines with excellent performance. Instead, computer modeling provides a potential way to bridge this gap. As the performance of such complex DNA machines will depend on the intricate dynamics of the operation mechanisms that occurs in three spatial dimensions, a model of DNA that naturally captures the subtle interplay between DNA structure, mechanics and thermodynamics has the potential to both explain motor behavior and to facilitate rational design and optimization.

The oxDNA model, which is a coarse-grained model of DNA at the nucleotide level (43–45), is particularly well-suited to meet this challenge. OxDNA has proven to be a powerful simulation tool that is capable of describing the physical properties of DNA that are relevant to DNA motors (46,47). In particular, oxDNA’s ability to describe the thermodynamics and dynamics of DNA hybridization (43,44,48,49) and displacement (9), the crucial role of geometry and stress in hybridization and melting reactions (50) (arXiv:https://arxiv.org/abs/1506.09008), the mechanics of single-stranded DNA (44), the structural and dynamic properties of DNA origami (45,51), as well as other large DNA nanostructures (52,53), lead us to believe that the model provides an ideal means to study the operation of the current motor system.

Here, we study the dynamics of the leg-placing reaction, which we have previously shown to be the yield-limiting step for our bipedal motor (34,35). We are primarily interested in the dynamics of the attachment of the walker’s leg to a foothold via a fuel strand, as well as on the dependency of the dynamics both on the step size and on its direction with respect to the origami track. The operation of the walker components has been carefully investigated by both experiments and molecular simulations. In the experiments the reaction yield was measured by single molecule fluorescence (54), while in the simulations the yield is obtained by computing the activation barriers for the reactions that determine motor efficiency. The simulations also allowed us to characterize the equilibrium structure of the origami in detail.

Three key experimental results, some unexpected and non-trivial, were largely reproduced by the oxDNA simulations. First, by sampling the ensemble of transition state geometries for the leg-placing reaction, the simulations accurately capture the overall dependence of the stepping probability on the foothold separation and predict the maximum possible motor step size (∼40 nm). Second, unexpected differences between experimental leg-placing yields for motors walking along the long and the short axes of the rectangular origami were explained by the preferential bending of the origami along the short axis. Third, the simulations suggest that the somewhat lower leg-placing yields for small steps (∼5 nm) compared to longer steps (∼12 nm) is due to secondary structure in the single-stranded sections of the walker.

## MATERIALS AND METHODS

### Motor design

**Figure 1.**
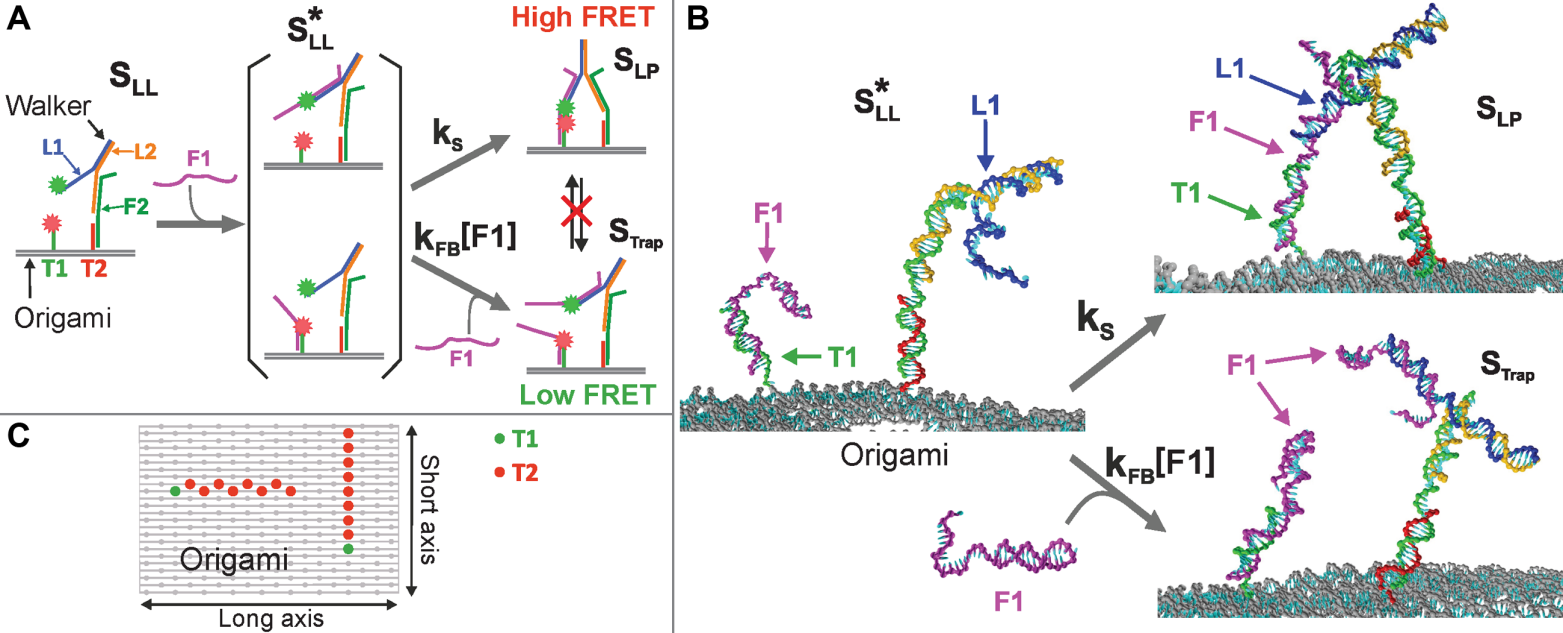
Schematics of the motor and the leg-placing reaction. (**A**) The motor consists of two footholds (T1 and T2) that are attached to a DNA origami, a walker that is made of two legs (L1 and L2) and two fuels (F1 and F2) that connect the walker to the footholds. The leg-placing reaction starts with L1 lifted (*S*_LL_). Introduction of F1 results in one of two intermediate states: ($S_\text{LL}^{*}$) followed by the formation of the leg-placed state (*S*_LP_) or the trapped state (denoted *S*_Trap_), which occur with rates *k*_s_ and *k*_FB_[F1], respectively. (**B**) Typical oxDNA configurations for the $S_\text{LL}^{*}$, *S*_LP_ and *S*_Trap_ states. (**C**) Schematic showing the alternative positions of the T1 and T2 footholds (green and red dots, respectively) on the rectangular origami. Adjacent footholds are designed to be roughly 5 nm apart, based on a simple origami model, where the rise per base pair is 0.34 nm and the width of the origami is 60 nm (from AFM measurements).

Our motor consists of a bipedal walker and a Rothemund-type DNA rectangular origami track (34,35). The bipedal walker is made up of two DNA strands called legs (L1 and L2), and the track consists of two DNA strands called footholds (T1 and T2) that branch out from the origami on the same side. The relevant states of the walker are shown schematically in Figure 1A. The walker and track are connected to each other by one or two DNA strands called fuels (F1 and F2 in the figure). Figure 1B shows typical oxDNA configurations for the walker in some of these states when attached to the origami track. The different designed positions for the T1 and T2 footholds on the origami that we considered are illustrated in Figure 1C; these allow different step sizes to be probed and the motor to walk along both the long and the short axes of the origami. Each experiment started with the walker standing on foothold-2 with leg-1 lifted. Only one foothold-2 was present in each experiment (see Figure 1C for alternative positions). The DNA sequences of the legs, footholds and fuels, as well as details regarding the origami track, are provided in the Supplementary Data and Table S1. For a detailed description of the entire walking cycle, which also includes the leg-lifting reaction (not studied here), see references (34,35).

### Mechanism of the leg-placing reaction

The leg-placing reaction is described in Figure 1A. Initially, the motor is in the leg-lifted state (denoted *S*_LL_), where one leg (L2) and a foothold (T2) are connected via a fuel strand (F2). The method for preparing the motor in this state is described in the Supplementary Data. The leg-placing reaction is initiated by introducing the F1 strand into the solution. This fuel may bind to the foothold (T1) or to the lifted leg (L1), forming two possible intermediate structures (both are denoted $S_\text{LL}^{*}$). This may be followed by binding of the fuel to the leg or to the foothold (L1 or T1, respectively) forming the leg-placed state (*S*_LP_) with a stepping rate *k*_s_ (see Figure 1A) (34,35). The stepping process ($S_\text{LL}^{*}\rightarrow S_\text{LP}$) is the reaction that is the focus of this work. Importantly, we have previously shown that another reaction that directly competes with the stepping reaction can also take place (34,35). In this reaction, the intermediate ($S_\text{LL}^{*}$) binds an additional fuel (F1) with a fuel-binding rate *k*_FB_[F1], forming a state called the ‘trapped state’ (*S*_Trap_). Because the fuels are, on the timescale of the experiments, irreversibly attached to the legs and footholds (by 16 and 18 bp, respectively), the trapped state is stable, blocks the formation of the *S*_LP_ state and hence hinders correct motor operation (34,35).

### Experimental leg-placing reaction yield

In our experiments, the fuel concentrations were much higher than that of the motors (see Supplementary Data). Therefore, for a pseudo first-order inter-molecular reaction, the rate of fuel binding depends linearly on the fuel concentration, i.e. *k*_FB_[F1] (34,35). By contrast, the rate of the intramolecular stepping reaction (*k*_s_) is independent of F1 concentration, but depends on the free-energy barrier for connecting an already attached fuel to the free leg or foothold. For simplicity, we assume that *k*_FB_ and *k*_s_ do not depend on whether the fuel strand F1 first binds to T1 or L1 (34,35). By incubating the motor and the fuel for long enough time before the measurement (for details, see the Supplementary Data) we ensure that the motor reaches either the *S*_LP_ state or the *S*_Trap_ state. Therefore, the leg-placing reaction yield, which is defined as the fraction of motors that reach the *S*_LP_ state, is the outcome of the kinetic competition between these two reactions, and is defined as:

(1) \begin{equation*} Y_\text{exp} = \frac{\left[S_\text{LP}\right]}{\left[S_\text{LP}\right]+\left[S_\text{Trap}\right]} = \frac{k_{\rm s}}{k_{\rm s}+k_{{\rm FB}}\left[\text{F}1\right]} \end{equation*}

where, [F1] is the fuel concentration.

### Measurement of the leg-placing reaction yields

**Figure 2.**
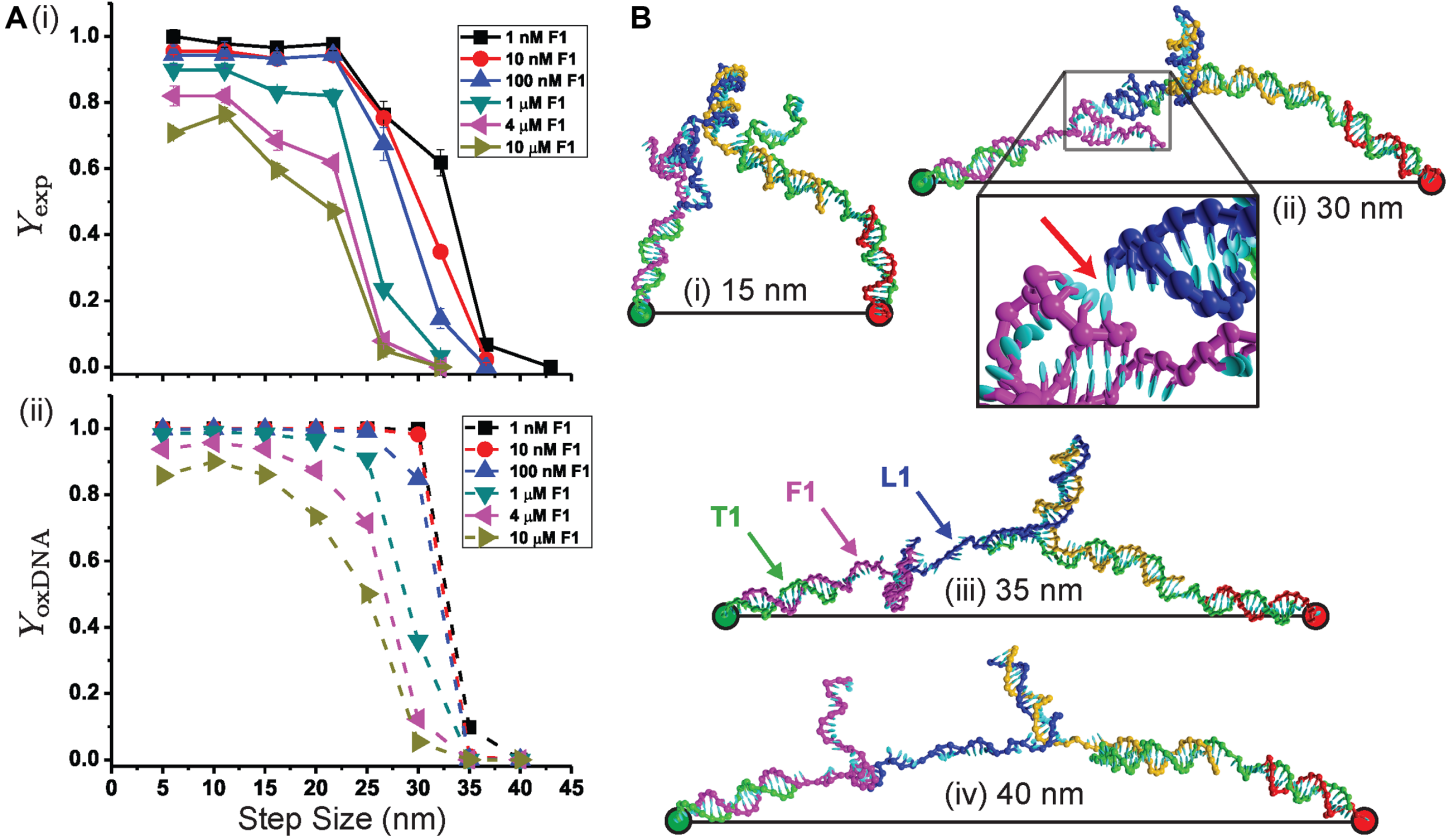
(**A**)(i) Measured experimental yield and (ii) the yield as determined from oxDNA simulations as a function of step size for different fuel concentrations. The step sizes in (i) correspond to the mean values measured in simulations of the free origami (Supplementary Table S2). (**B**) Example oxDNA transition state configurations (with 1 bp between fuel F1 and leg L1) illustrating the increasingly stretched nature of the walker at larger step sizes. The red arrow in (B)(ii) indicates the position of a leg-placing base pair.

To measure the relative fractions of motors in the *S*_LP_ and *S*_Trap_ states, we utilized diffusion-based single-molecule Förster resonance energy transfer (FRET) (34,54). L1 and T1 were labeled with donor and acceptor fluorophores (Figure 1A) such that the system yields clearly distinguishable low- and high-FRET values for the two states, respectively (for a detailed description of the measurement technique and data analysis procedures, see the Supplementary Data and Figure S1). The experimental yields are depicted in Figure 2A as a function of the step size and fuel concentration, and will be discussed in detail below together with the simulation results.

### Coarse-grained simulations of the leg-placing yields

In order to compare experimental results with oxDNA simulations, we simulated the walker and the origami at the same environmental conditions as in the experiments. Details of the oxDNA model and the simulation algorithms used can be found in the Supplementary Data. To measure the yield in the simulations, we first computed free-energy profiles for both the reactions illustrated in Figure 1B, namely the leg-placing reaction (initiated in a state with the fuel attached to the foothold), and the binding of a fuel molecule to L1. Note that for computational simplicity, we only considered one of the possible intermediate states, as we expect to a good approximation *k*_s_/*k*_FB_ to be the same for both. Note also that because of the large size of the walker and origami system, when we computed the relevant free-energy profiles, we did not use the oxDNA model to represent the origami, but instead modeled the origami as a repulsion plane and used harmonic traps to hold the two foothold strands at the relevant separations on the plane (see the Supplementary Data for more details).

**Figure 3.**
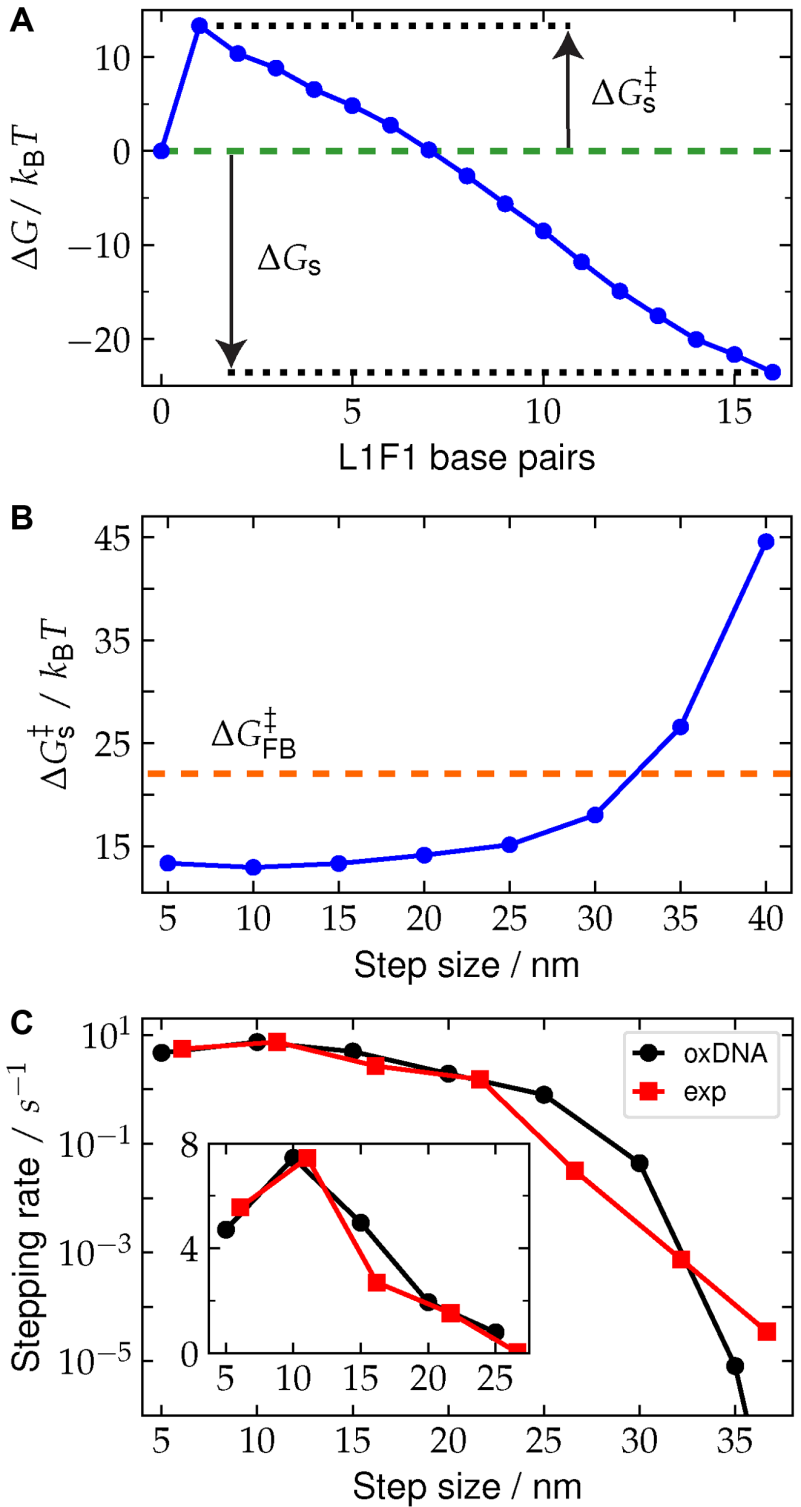
(**A**) Example free-energy profile for leg placement for a foothold separation of 15 nm, where $\Delta G_s^\ddagger$ is the activation free energy, and Δ*G*_*s*_ is the free energy change for the reaction. (Additional free-energy profiles for different step sizes and for the fuel-binding reaction are shown in Supplementary Figure S5). (**B**) Dependence of $\Delta G_s^\ddagger$ on step size. The horizontal dashed line shows the value of $\Delta G_{FB}^\ddagger$ at [F1] = 10 nm. (**C**) The experimentally determined stepping rates (red squares) are plotted as a function of step size. The oxDNA predicted relative rates (black circles) are positioned to match with the experimentally determined value at 12 nm.

An example free-energy profile for leg placing is shown in Figure 3A. There is a large free-energy barrier to initiating the first base pair between the fuel and the walker due to the loss of conformational entropy that this involves, followed by a steep, downhill slope as the remaining base pairs zip up. From such free-energy profiles both the net change in free energy and the activation free energy for the processes can be found, the latter allowing us to estimate the kinetic yield measured experimentally. Although we do not measure the rates *k*_s_ and *k*_FB_ directly, it is reasonable to assume that the relative rates are dominated by the effects of the activation free energies, thus giving

(2) \begin{equation*} Y_\text{oxDNA} \approx \frac{e^{-\Delta G_s^\ddagger / k_\text{B} T}}{ e^{-\Delta G_s^\ddagger / k_\text{B} T} + e^{-\Delta G_{{\rm FB}}^\ddagger \left([\text{F}1]\right) / k_\text{B} T}} \end{equation*}

where, *k*_B_ is the Boltzmann constant and *T* is the temperature. For additional details regarding the calculation of the yield as well as the simulation details, see the Supplementary Data.

## RESULTS AND DISCUSSION

### Dependency of the leg-placing yields on step size

Figure 2A shows the experimental and simulated leg-placing reaction yields for walking along the long axis of the origami for 1 nm to 10 μm fuel concentrations. The two plots look very similar showing that the simulations can well describe the observed behavior and, hence, provide important microscopic insights into its origins. The most dramatic feature of the data is a rapid decrease in the yield at near to 30 nm as the system crosses over between a regime where leg placing dominates at short steps to one where the yield is essentially zero at large step sizes.

From Figure 3B it can be seen that the sharp crossover reflects the rapid increase in the free-energy barrier for leg placing with the center of the transition in the simulated data occurring when $\Delta G_s^\ddagger \approx \Delta G_{FB}^\ddagger$. The oxDNA leg-placing transition state configurations illustrated in Figure 2B provide a simple explanation of this rise. For step sizes in the range 5–20 nm the walker, which possesses a substantial amount of flexibility, does not have to stretch that much to put its foot down (see also Supplementary Figure S2 for configurations of the fully-bound walker). At these step sizes, encounters between the walker and foothold are relatively common. However, as the step size increases, the walker must increasingly stretch farther to reach the fuel, making it more difficult to form the initial leg-placing base pairs. At the 40 nm step size, the walker is still capable of putting its foot down by nearly completely stretching out and lying almost parallel with the origami surface. However, because the activation barrier to initiate leg-placing base pairs is so large, the trapped state is effectively the only relevant state.

As the fuel concentration increases, trapping by binding of multiple fuels of course becomes more likely, and has two main effects on the yield curves. First, the point at which the large decrease in yield occurs moves to lower step size, but only relatively slowly because of the sharp rise in $\Delta G_{s}^\ddagger$ in the relevant step size range (Figure 3B). Second, at smaller step sizes the yield starts to decrease from the close to 100% yield seen for the lowest concentrations. As the activation barrier only depends relatively weakly on step size in the range 5–20 nm (it varies by about 1–2 *k*_B_*T*) the effect of concentration is more uniform, with the small differences in response reflecting the variations in $\Delta G_s^\ddagger$. We do see, however, somewhat more pronounced yield decreases in experiment than simulations, but we note that these can be explained by relatively small errors in the activation-free energies (∼2 *k*_B_*T* or less). Given some of the simplifications of our modeling of the motor operation, the overall agreement is very good.

One intriguing feature of the behavior for short steps is that the yield in experiment and simulations drops slightly for the 5 nm step compared to 10 nm for very high fuel concentrations. Although the effect is small, the origin of this effect in the simulations is clear, as it disappears when the single stranded sections are not allowed to form any secondary structure. In particular, one of the predominant hairpins that forms in the F1 fuel when bound to T1 significantly reduces the likelihood that the end of the fuel will be close to the T1 attachment point (Supplementary Figure S9), as can be seen from the illustrative configurations in Supplementary Figure S10. The hairpin thus makes it harder to form the first base pair at the shortest step sizes, and reduces the yield. Note that we did not intentionally design this hairpin (the leg and foothold sequences follow the designs of the original motors) (22,34–36). Supplementary Figure S4 shows that the differences between motors with and without secondary structure in the ssDNA segments of the motor do not significantly change the yields at other step sizes. In the original motor designs, the leg and foothold sequences were originally optimized to minimize secondary structures (using NUPACK (55) or mfold (56)); however, transient hairpin structure was revealed by oxDNA. As we learn here, a better motor should not include such secondary structures.

Finally, we calculated the stepping rates for different step sizes, which are shown in Figure 3C. Since the stepping yield depends on the competition between stepping and fuel-binding reaction rates (Equation 1), the experimentally measured yields can be combined with a previously determined fuel-binding rate (35) ($k_{{\rm FB}} = 2.3\times 10^{5}\, \text {M}^{-1}\, \mathrm{s}^{-1}$) to obtain the stepping rates. With oxDNA, we estimated the stepping rate at different step sizes relative to the 10 nm size, by noting that the relative rates are primarily determined by the difference in the activation energies, $k_{\rm s}(d)/k_{\rm s}(10\, \mathrm{nm}) = \exp (-(\Delta G_{{\rm s}}^\ddagger (d)-\Delta G_{{\rm s}}^\ddagger (10\, \mathrm{nm}))/ k_\text{B} T)$ (see the Supplementary Data). Figure 3C shows for step sizes 5–25 nm that the stepping rates are ∼1–10 s^−1^. Clearly, further increasing the step size reduces the stepping rate significantly. For example, at 35 nm the stepping rate is reduced to 3.5 × 10^−5^ s^−1^, which is about five orders of magnitude slower than *k*_s_ for the short step sizes. Overall, the oxDNA predicted relative rates are in good agreement with the experimentally obtained rates, with some differences apparent for larger step sizes that we again attribute to small errors in the activation barrier calculations.

### Comparison of walking along the long and short origami axes

**Figure 4.**
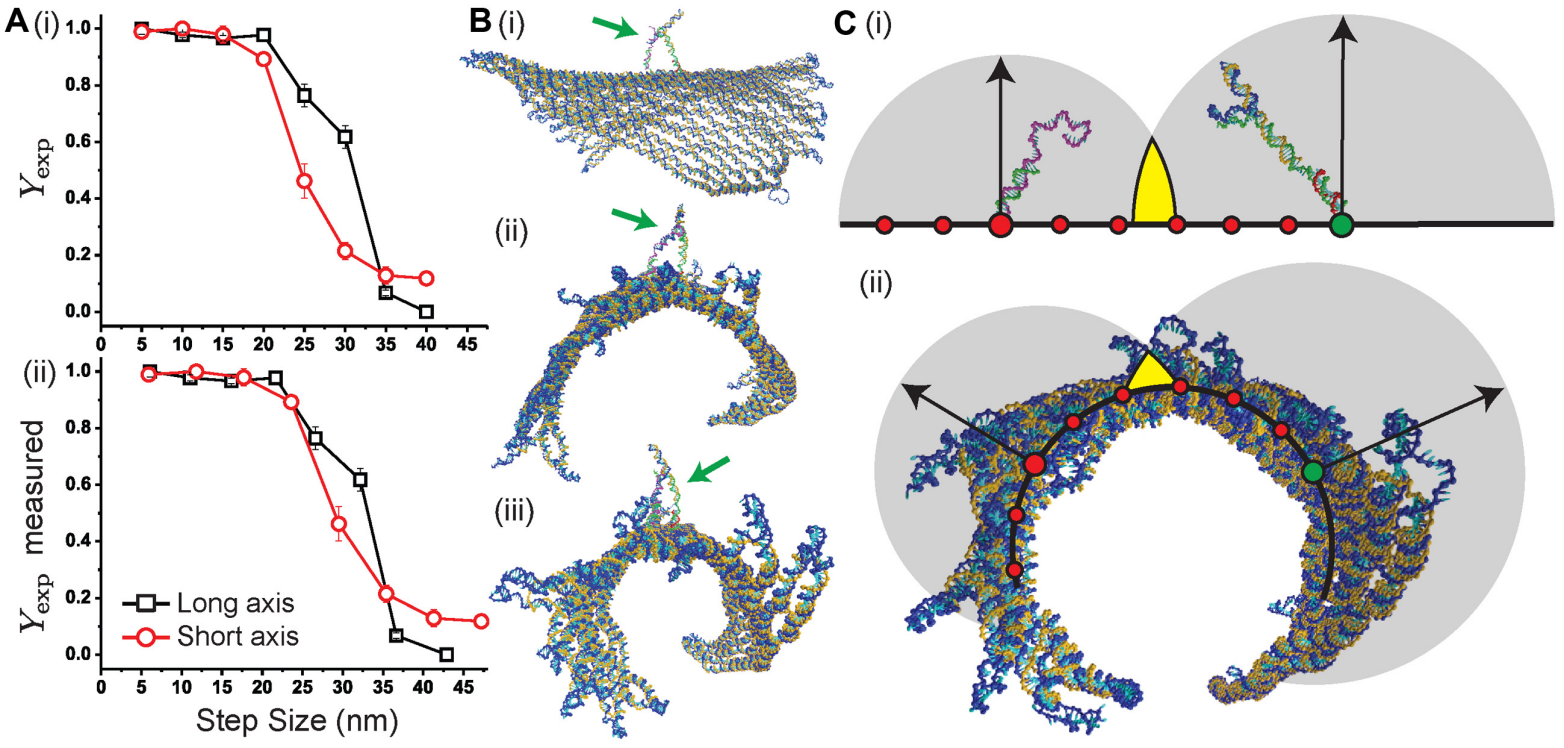
(**A**) The experimentally measured yields are shown as a function of (i) the designed step size in Figure 1C, and (ii) the measured step size based on the results from simulations of the origami. The concentration of F1 in the experiments was 1 nm. (**B**)(i-iii) Typical configurations of the curled up origami with the walker (denoted by the green arrow) positioned on the long axis. (**C**) Schematic figures showing (i) the walker (blue-orange) with one foot up and the other down on a flat surface for a step size ∼32 nm, and (ii) an axial view of an origami configuration curled up into a tubular structure, with short axis foothold positions denoted by circles. The step size between the larger red and green circles in (C)(ii) is the same as in (C)(i). The gray circles in both (i) and (ii) represent the regions of space well-sampled (95% of configurations have their terminal base within the sphere) by the unbound section of walker (blue) and the foothold-bound fuel (purple) when secondary structure was forbidden in the simulations. Arrows represent the radii of the spheres and yellow color indicates the overlap between the spheres.

Figure 4A(i) shows a comparison of the experimental leg-placing yields for walking along the long and the short axes of the origami, both plotted as a function of the designed step size. Although similar, there are two clear differences. First, the sharp decrease in yield occurs at smaller step size for the short axis then for the long axis. Second, unlike for the long axis, the yield does not continue to decrease to near zero at the longest distances but seems to plateau to a value of about 10%. These difference in yield between the two axes are clearly telling us something about the nature of the origami surface.

**Figure 5.**
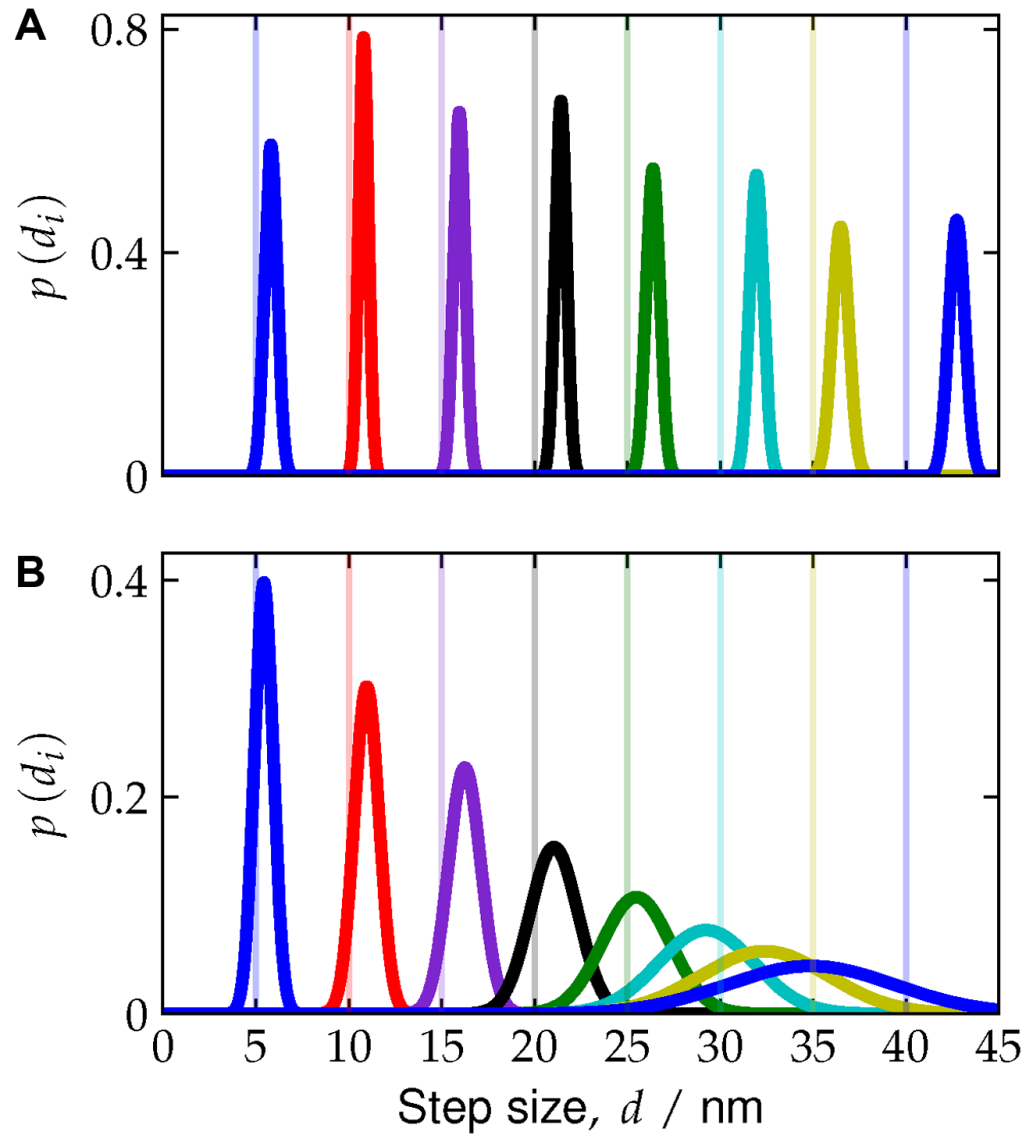
The distributions for step size *d* on the (**A**) long axis and (**B**) the short axis. In both figures the step size is the measured distance between a foothold position (red dots in Figure 1C) and a reference position (green dot in Figure 1C). Vertical lines denote the designed foothold separations.

To understand these discrepancies, we simulated the complete origami with oxDNA and characterized its equilibrium structure. For comparison, we also characterized the origami when placed onto a surface that renders it mostly flat (see the Supplementary Data). Figure 4B shows representative configurations of the origami with the walker positioned along the long axis. We also measured the separation of the attachment points for foothold-1 and foothold-2 (Figure 1C) for both axes. The results are shown in Figure 5A and B for the long and short axes, respectively. Comparable results for the flat origami are shown in Supplementary Figure S8.

Figure 5A clearly shows that the distributions for the different foothold positions along the long axis are narrow and nearly evenly separated by roughly 5.9 nm, and have average values comparable to that of the flat origami (see Supplementary Table S2). Thus, the track along the long axis of the origami is stiff and relatively straight. The short axis distributions for the smallest step sizes (5 and 10 nm), shown in Figure 5B, are narrow, and comparable to those quantities for the flat origami. However, the distributions are significantly broader for larger foothold separation and have values that are significantly shorter than those measured for the flat origami (Supplementary Table S2). This difference is due to the origami being much more flexible along the short axis and having significant curvature.

This curvature is apparent in the configurations shown in Figure 4B and C; the origami has a preferred bending direction and rolls up into a somewhat twisted tubular structure. In particular, the origami is always seen bending away from the side on which the nicks are, thus the walker remains on the convex side. Note that the curled up structure means that for the longer step sizes along the short axis, the results in Figure 5B represent straight-line distances that pass through the curved structure rather than along its surface.

The twisted nature of the current origami is unsurprising since the effective designed pitch in the origami (10.67 bp per turn) is greater than that for real DNA (about 10.5 bp per turn) (57,58). For a 2D sheet, this twist naturally is coupled to bend, and, since the two faces of the origami are different, the two possible modes of bending are distinct. The bending direction predicted by oxDNA is also consistent with the direction predicted by CanDo (see also Supplementary Figure S11) (59).

To check that some of the differences in yield are not simply due to the initial simplified method of measuring the step size, Figure 4A(ii) shows the same data for the experimental yields as in Figure 4A(i), but plotted using the step sizes for both axes measured in the simulations of the origami. Since the long axis remained mostly flat in the simulations, each step size was taken to be equal to oxDNA’s average value for the separation of the footholds (Supplementary Table S2). However, for the short axis we estimated the step size by adding up the contributions from measuring the average separation between neighboring footholds, which comes to be about ∼5.9 nm per foothold pair (see Supplementary Table S3). Although these adjustments shift the step sizes to slightly larger values, this occurs for both axes and only slightly reduces the differences in yield between the two axes.

Instead these differences stem from the preferred bending direction of the origami. In particular, the early drop in yield along the short axis can be understood by considering the configurations in Figure 4C, which show the walker system at ∼32 nm step size on a flat surface (Figure 4C(i)), and a schematic of the short axis foothold locations on the curled up origami (Figure 4C(ii)). The overlap between the accessible extensions of the walker and the toehold with F1 fuel bound is clearly less for the convex surface, thus increasing the activation barrier for the formation of the first base pair and causing the yield to decrease. The opposite effect would be expected if the walker was on the concave side of the origami.

Finally, for the step sizes larger than ∼30 nm, because of the bending of the origami, the L1 section of a walker may instead reach to T1 from the opposite direction. This is why we see a small fraction of yield persist even for the ∼45 nm step size, which is absent for the stiff long axis for which there are no alternative paths.

## CONCLUSION

To conclude, we have thoroughly characterized the key step for the operation of a DNA walker, namely the leg-placing reaction, using single-molecule fluorescence techniques with a particular emphasis on the dependence of the yield on step length. Furthermore, we have shown that coarse-grained modeling accurately captures the interplay of structure, mechanics and thermodynamics in a way that explains a number of non-trivial features of the observed behavior, highlighting the potential utility of such modeling to guide the design of improved motors, both through an enhanced understanding of the physical principles underlying the motor operation and by pre-screening the behavior of putative designs.

For the specific walker design, we have considered here, we found that the motor can efficiently operate over a wide range of step sizes due to the flexibility of the walker, but with a strong decrease in yields for steps beyond about 30–40 nm (depending on fuel concentration) associated with the rapid increase in the free-energy barrier due to the walker having to extend itself to a very entropically unlikely state to initiate leg placing. We have also found that the motor operation is sensitive to the structural details of the origami substrate due to its anisotropic mechanical properties and the preferred curvature of the current design —the latter effect could be reduced by tuning the effective pitch length of DNA in the origami.

How do the legs and footholds lengths change the dependency of stepping yields on step sizes? One can speculate that the maximum possible step size will increase as the lengths of footholds and legs are made longer. However, for intermediate and short step sizes, increasing these lengths will also increase the available search-space volume accessible to the walker, which will increase the activation barrier to leg-placing and decrease the leg-placing rates, and therefore also the stepping yields. The foothold and walker legs in the current design also all have lengths well below the persistence length for dsDNA. As a result, these sections are quite stiff, however, longer footholds and legs will inevitably increase the overall degree of flexibility present in the motor system, further complicating the dependency of the walking efficiency on step size. In future studies, we aim to answer this non-trivial question using coarse-grained simulations and experiments.

Finally, what lessons can be learned for the optimization of DNA walkers? First, in the current motor design, which has four different footholds that are separated 12 nm from each other (34–36), a small fraction of the motors may walk in the wrong direction (instead of one step forward, it takes three steps backward). To ensure that the walkers only move in the intended direction, we suggest using a linear track with six different footholds that are separated 12 nm from each other. We are currently testing a motor with two sets of six footholds organized linearly (a total track length of ∼140 nm). Second, to avoid walkers stepping over to the opposite side we suggest adapting methods that reduce origami bending. For example, origami bending can be reduced by tuning the effective pitch length of the origami DNA (4,60,61). Alternatively, origami made of two or more layers, which are known to be stiffer than a single layer (5,38,40,62), can also be used to prevent bending. In addition, immobilizing the origami onto a stiff surface, such as a cover slide, could also be used to prevent bending. At last, our study shows that it is essential to overcome the formation of a trapped state, which is the key bottleneck in the current design limiting motor performance. For that purpose, we are developing an alternative walking strategy in which we are able to avoid the formation of the trapped state entirely by switching the order in which fuel and anti-fuel strands are provided to the system, and by redesigning the strands. We have obtained preliminary results suggesting that these improvements yield significantly better performing motors.

## DATA AVAILABILITY

The simulation code is available online (https://dna.physics.ox.ac.uk.). Data associated with the simulations is available at the Oxford University Research Archive (DOI:10.5287/bodleian:w4ZwVr6Jg).

## Supplementary Material

Supplementary Data
