# Supplementary material for: DNA bipedal motor walking dynamics: an experimental and theoretical study of the dependency on step size
Source: Nucleic Acids Res. 2017 Dec 27;46(3):1553–61. doi: 10.1093/nar/gkx1282 (PMC5814849; doi:10.1093/nar/gkx1282)
Supplement: Supplementary Data [file gkx1282_supp.zip › gkx1282_Supp.pdf]

Supplementary Data for "DNA Bipedal Motor Walking Dynamics: An Experimental and Theoretical Study of the Dependency on Step Size"

Dinesh C. Khara*,[1] John S. Schreck*,[2, a)] Toma E. Tomov,[1] Yaron Berger,[1] Thomas E. Ouldridge,[3] Jonathan P. K. Doye,[4, b)] and Eyal Nir[1, c)]

[1)] *Ben-Gurion University of the Negev, Beer-Sheva and the Ilse Katz Institute for Nanoscale Science and Technology, 8410501, Israel*

[2)] *Department of Chemical Engineering, Columbia University, 500 W 120th St, New York, NY 10027, USA*

[3)] *Department of Bioengineering, Imperial College London, South Kensington Campus, London SW7 2AZ, United Kingdom*

[4)] *Physical and Theoretical Chemistry Laboratory, Department of Chemistry, University of Oxford, South Parks Road, Oxford OX1 3QZ, United Kingdom*

Contents

SI. Experimental Information 1
- A. Motor Preparation and Design 1
  - 1. DNA Labeling 1
  - 2. Annealing 1
  - 3. Origami Purification 1
  - 4. Motor Design 2
- B. Single Molecule Fluorescence Experiment 2
  - 1. Preparation of the Motor for Single Molecule Experiments 2
  - 2. Data Analysis and Determination of Stepping Yields 2
  - 3. Sample Chamber 2
  - 4. Single-Molecule FRET/ALEX Optical Setup 2

SII. Additional Simulation Details and Results 2
- A. OxDNA Model 2
- B. Simulation Methods 4
  - 1. Thermodynamics: Virtual Move Monte Carlo 4
  - 2. Thermodynamics: Umbrella Sampling 5
  - 3. Molecular Dynamics 5
  - 4. Modeling the Origami Track in VMMC Simulations 5
  - 5. Simulations of the Origami on a Surface 6
- C. Simulation Protocols 7
  - 1. Thermodynamics 7
  - 2. Calculation of the Yield 8
  - 3. Calculation of Average Step Sizes 8
  - 4. Calculation of Walker and Foothold Ranges 9
- D. Supplemental Results 9
  - 1. Computed Free-energy Landscapes and Yields 9
  - 2. Range Calculation Results 9
  - 3. CanDo Comparisons 12
  - 4. Movies 12

a) Electronic mail: jsschreck@gmail.com
b) Electronic mail: jonathan.doye@chem.ox.ac.uk
c) Electronic mail: eyalnir@bgu.ac.il

SI. Experimental Information

A. Motor Preparation and Design

1. DNA Labeling

HPLC-purified DNAs were purchased (IDT, Coralville, LA) with a C6 dT internal amino modifier (iAmMC6T) at designed locations, labeled with ATTO-550 or ATTO-647N (donor and acceptor, respectively, ATTO-TECH), and HPLC purified (reverse-phase C18, Amersham Bioscience).

2. Annealing

Annealing was performed in 50X TAE buffer (TAE; 40 mM Tris, 1 mM EDTA, 40 mM acetic acid, pH 8.0) and 12 mM MgAc2 in a 50-μL volume. The procedure was as follows: heating to 95 °C for 10 min, cool to 84 °C at 1 °C/min, cool to 70 °C at 1 °C/5 min, cool to 40 °C at 1 °C/15 min, and cool to 20 °C at 1 °C/10 min. The annealing solution contained 2 nM scaffold (New England BioLabs), 5-fold excess staples (edge staples were omitted) and 20-fold excesses of footholds, fuels, and legs.

3. Origami Purification

The origami was purified using a size-exclusion column.[1] The column was hand-packed with 750 μL of liquid resin (Sephacryl S-300 HR, dsDNA cut-off of 118 bp; GE Healthcare, Little Chalfont, UK) and then spun at 1400 g for 2 minutes to result in 500 μL dry volume. The column was equilibrated by washing three times with 500 μL of 1X TAE buffer with 4 mM MgAc2 and centrifuging for 2 minute at 1400 g to remove excess buffer. Origami sample (50 μL) was added to the column and centrifuged at 1400 g for 4 minutes. This procedure was repeated three times to achieve complete removal of excess staples.

4. Motor Design

The motor design is based on our previously published motors[2,3] with minor modifications and the origami track is based on the Rothemund rectangular origami.[4] The DNA sequences are given in Table SI.

B. Single Molecule Fluorescence Experiment

The sm-FRET/ALEX experiments were carried out on an optical setup built in-house. For detailed discretion of the optical setup, data analysis and definitions of FRET (E) and fluorophore stoichiometry (S) see our previous publications.[2,3]

1. Preparation of the Motor for Single Molecule Experiments

To minimize walker dissociation during purification the motor was prepared such that the walker legs were attached to footholds T2 and T3 via fuels F2 and F3, respectively (Figure S1a, left panel). After purification, antifuel-3 strands (AF3) were added to the motor solution (50 nM) to lift L1 from T3 (Figure S1a, middle panel) and left to react for 3 hours. A small volume of the motor solution (1μL) was diluted into the measurement buffer (10 pM motor concentration). The measurement buffer was comprised of 10 mM Tris (pH 8.0), 1 mM EDTA, 10 μg/mL BSA (Sigma-Aldrich) to reduce sample sticking, 500 mM NaCl, and 1mM Trolox (Sigma-Aldrich) to reduce fluorophore photo-bleaching and photo-blinking. A certain concentration of F1 (1 nM to 10 μM) was added to the solution and the sample was left to react for 1-10 hours to allow completion of the leg placing reaction (Figure S1a, right panel).

2. Data Analysis and Determination of Stepping Yields

Leg-1 (L1) was labeled with donor fluorophore (ATTO-550) and foothold-1 (T1) was labeled with acceptor fluorophore (ATTO-647N). When L1 was lifted (S_{LL}) the FRET efficiency was $E \approx 0.3$ and when L1 was placed on T1 the FRET efficiency was $E \approx 0.8$ (SLP, Figure S1b). Stepping yield was calculated by dividing the number of motors with high E values ($0.7 < E < 1.0$) to that of motors with high plus low ($0.2 < E < 0.4$) E values. To distinguish between intact motors and residual motors not attached to origami track using the ALEX technique, an additional ATTO-647N fluorophore was labeled with the origami (far from the walker, Figure S1). This additional acceptor is far from the donor (distance > 40 nm) and, therefore, does not contribute to the FRET process and the E values. This yields motors with stoichiometry ratio between $0.25 < S < 0.4$ (indicating one donor and two acceptors). Therefore, calculation of the stepping yield was based only on events (photon bursts) with stoichiometry ratio between these values (Figure S1b). Data was accumulated for 45 min for each data point (Figure 2a and 4a, main text).

3. Sample Chamber

Measurements were done on a KOH-treated coverslip that was sonicated (15 min) in 1 M KOH solution, thoroughly washed with distilled water, and dried with air. To prevent solution evaporation, the coverslip was sealed with silicone isolation sheet (Sigma-Aldrich), and, after sample deposition ($\approx$ 40 μL), an upper coverslip was gently placed on the silicone to seal the chamber.

4. Single-Molecule FRET/ALEX Optical Setup

The sm-FRET/ALEX experiments were carried out on an in-house built optical setup.[2,3,5] In brief, a green CW laser beam (532 nm, CL532-025-L, Crystal Laser) was aligned/misaligned into a single-mode fiber using an acousto-optic modulator (AOM; R23080-2-LTD, Neos Technologies), alternating with a red diode laser (640 nm, 1069417, Coherent) that was electronically switched on and off. The AOM and the red laser were computer controlled with a 25-μs alternation cycle. The laser beams were combined by a dichroic mirror (Z532RDC, Chroma) and coupled into a single-mode fiber (P1-460A-FC-2, Thorlabs). The laser intensities were tuned such that the doubly labeled species yielded $S \approx 0.5$ (110 μW for the green laser and 42 μW for the red laser, measured after the fiber while alternating). After collimation (objective PLCN10X/0.25, Olympus), the combined green and red beams were introduced into an inverted microscope (IX71, Olympus America) and focused about 70 μm inside the sample solution by a water-immersion objective (NA 1.2, 60X, Olympus America). The emitted fluorescence was separated from the excitation light by a dichroic mirror (ZT532/638RPC, Chroma), focused into a 100-μm pinhole (P100S, Thorlabs), re-collimated, split by a second dichroic mirror (FF650-Di01, Semrock), filtered (band-pass filter, FF01-580/60, Semrock, for the donor channel and a long-pass filter BLP01-635R, Semrock, for the acceptor channel), and focused into two single-photon avalanche photodiodes (SPAD; SPCM-AQRH-13, Perkin-Elmer Optoelectronics). The electronic signals (TTL) of the two SPADs were recorded as a function of time by a 12.5-ns resolution counting board (PCI-6602, National Instruments) and analyzed using in-house prepared Labview acquisition software.

SII. Additional Simulation Details and Results

A. OxDNA Model

In oxDNA, a single strand of DNA is modeled as a chain of 3D rigid nucleotides. The effective potential energy of a configuration in oxDNA is given by a sum of pairwise interactions between nucleotides and includes terms for hydrogen-bonding, cross-stacking, coaxial stacking, nearest-neighbor stacking, excluded volume and backbone chain connectivity. Base-pairing interactions obey Watson-Crick specificity (i.e. A–T or G–C pairs), but other interactions such as Hoog-

Strand name	Original strand	Sequence (5′ → 3′)
		**Legs**
L1		GGCTGGTTTCTGCTCTCTAGTTCGCGAGGTGCAATCTCCTATC(ATTO-550)
L2		CGATGGTGTCTAGATCACTGTTCCTAGAGAGCAGAAACCAGCC
		**Fuels and Anti-Fuels**
F1		AGTAACCAAAGTACAGCACTGTTTTAGGAGATTGCACCTCCAATTTACCC
F2		GTAGCGTGACTGTTACGGTGATCTAGACACCATCGTTGAAACGCTACTGCCCATACA
F3		ACAGAGTCCGATGTGGAAGTCTTTTAGGAGATTGCACCTCCCATGTTAGCTCCACAT
AF3		GCTAACATGGGAGGTGCAATCTCCTAAAAGACTTCCACATCGGACTCTGT
		**Footholds - Long axis**
T1	r-7t10e	GCTGT(ATTO-647N)ACTTTGGTTACTGAATTTTTGCGCAGAAAACGAGAATGAATGTTTAG
T2 (6.07 nm)	r-5t8f	AAAGATTCAGGGGGTAATAGTAAACCATAAATTTTTGTATGGGCAGTAGCGT
T2 (11.04 nm)	r-5t12e	CAATAAATACAGTTGATTCCCAATTTAGAGAGTTTTGTATGGGCAGTAGCGT
T2 (16.19 nm)	r-3t8f	CATTCAACGCGAGAGGCTTTTGCATATTATAGTTTTGTATGGGCAGTAGCGT
T2 (21.66 nm)	r-3t12e	TCAATTCTTTTAGTTTGACCATTACCAGACCGTTTTGTATGGGCAGTAGCGT
T2 (26.61 nm)	r-1t8f	GGAATTACTCGTTTACCAGACGACAAAAGATTTTTTGTATGGGCAGTAGCGT
T2 (32.17 nm)	r1t10f	CCTAATTTACGCTAACGAGCGTCTATATCGCGTTTTGTATGGGCAGTAGCGT
T2 (36.66 nm)	r-1t10e	TTTTAATTGCCCGAAAGACTTCAATTCCAGAGTTTTGTATGGGCAGTAGCGT
T2 (42.93 nm)	r1t12e	CTAATTTATCTTTCCTTATCATTCATCCTGAATTTTGTATGGGCAGTAGCGT
T3	r1t8e	TCCACATCGGACTCTGTTTTGCAATAGCGCAGATAGCCGAACAATTCAACCG
		**Footholds - Short axis**
T1	r7t18f	GCTGT(ATTO-647N)ACTTTGGTTACTTTTGAGCAAAAACTTCTGAATAATGGAAGAAGGAG
T2 (5.91 nm)	r7t14f	AGAGGCATAATTTCATCTTCTGACTATAACTATTTTGTATGGGCAGTAGCGT
T2 (11.82 nm)	r7t12f	TCATTACCCGACAATAAACAACATATTTAGGCTTTTGTATGGGCAGTAGCGT
T2 (17.73 nm)	r7t10f	CTTTACAGTTAGCGAACCTCCCGACGTAGGAATTTTGTATGGGCAGTAGCGT
T2 (23.64 nm)	r7t8f	TTATTACGGTCAGAGGGTAATTGAATAGCAGCTTTTGTATGGGCAGTAGCGT
T2 (29.55 nm)	r7t6f	CCGGAAACACACCACGGAATAAGTAAGACTCCTTTTGTATGGGCAGTAGCGT
T2 (35.46 nm)	r7t4f	TGAGGCAGGCGTCAGACTGTAGCGTAGCAAGGTTTTGTATGGGCAGTAGCGT
T2 (41.37 nm)	r7t2f	TGCTCAGTCAGTCTCTGAATTTACCAGGAGGTTTTTGTATGGGCAGTAGCGT
T2 (47.28 nm)	r7t0g	TATCACCGTACTCAGGAGGTTTAGCGGGGTTTTTTTGTATGGGCAGTAGCGT
T3	r5t6f	TCCACATCGGACTCTGTTTTAATCACCAAATAGAAAATTCATATATAACGGA

TABLE SI. Sequences of the DNA strands.

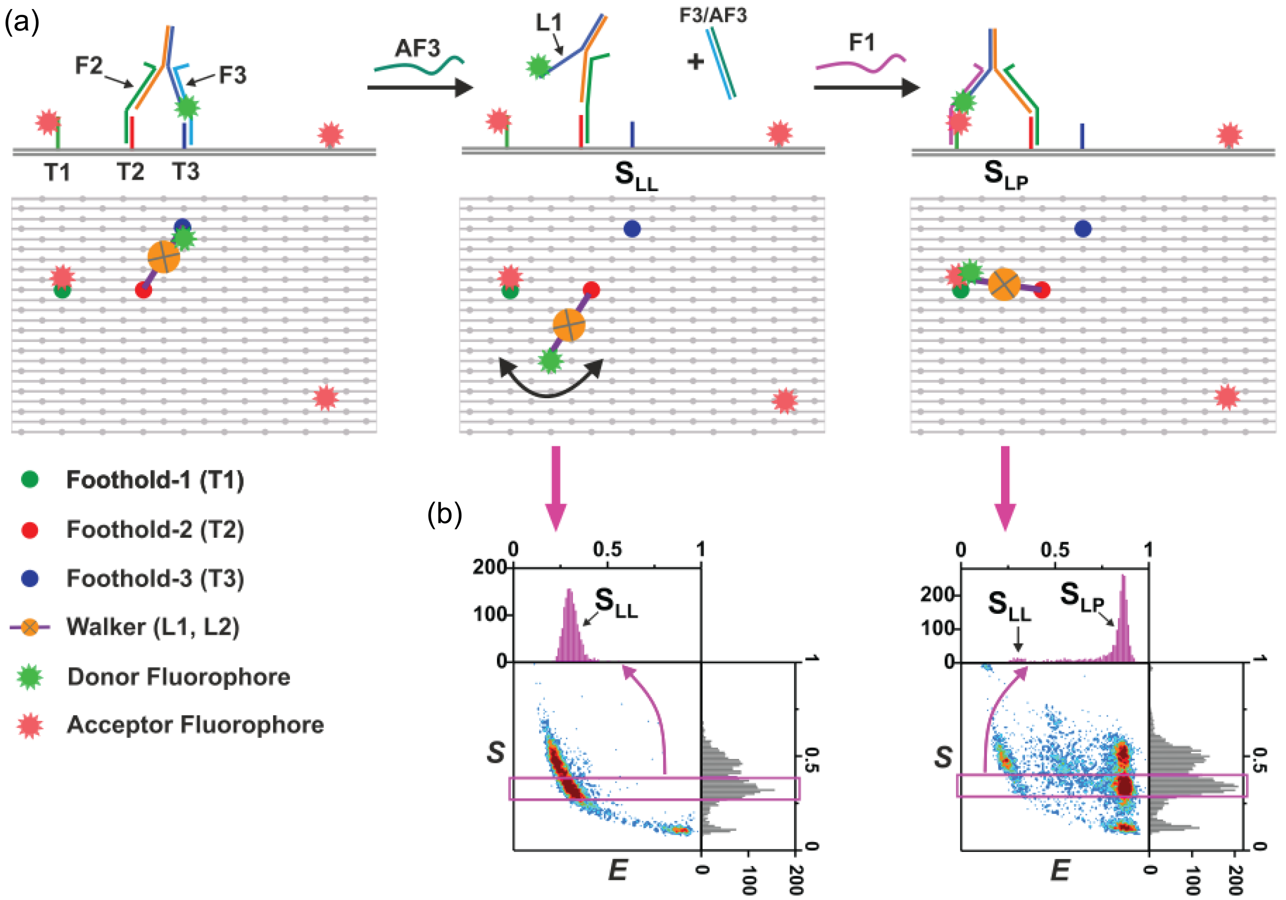

FIG. S1. Single molecule measurement. (a) Motor design. The motor was annealed with the walker standing on footholds T2 and T3 (left panel). After annealing and purification L1 was lifted from T3 by removing F3 using AF3 (middle panel, S_{LL}). Fuel-1 (F1) was added to the solution, and depending on the stepping yield, some L1 legs were attached to T1 footholds, forming leg placed state (S_{LP}, right panel). (b) Typical two-dimensional E/S histogram and E and S projections measured before and after introduction of F1 (left and right panels, respectively).

steen pairs are excluded from the model. Details of the interactions contributing to the oxDNA potential can be found elsewhere,[6,7] and simulation code for oxDNA can be downloaded from the oxDNA website.[8]

In the simulations we closely mimic the experimental conditions in which the motor system was investigated. To achieve this we use the sequence-dependent parametrization of "oxDNA 2.0,"[6,9] where the strengths of hydrogen bonding associated with base pairing and the stacking interactions are dependent on the bases involved, as well as an ionic salt concentration of $[Na^+] = 0.5\,\mathrm{M}$, where the electrostatic properties are strongly screened. We also ran all simulations at 22°C. The values for salt and temperature match those used in the experiments.

B. Simulation Methods

1. Thermodynamics: Virtual Move Monte Carlo

Simulations of the motor can be made more efficient by using the Virtual Move Monte Carlo (VMMC) algorithm proposed by Whitelam and Geissler. VMMC allows for collective motion of particles using cluster moves.[10] Specifically, we use the variant presented in the appendix of reference 10. Initially a nucleotide is selected, and a move is chosen at random. The nucleotide's neighbors are then added to a co-moving 'cluster' with probabilities determined by the energy changes that would result from the move. As a consequence, multiple nucleotides tend to move at once. To use VMMC, we must select 'seed' moves of a single nucleotide. For all VMMC simulations reported here, the seed moves were:

- Rotation of a nucleotide about its backbone site, with the axis chosen uniformly on the unit sphere and the angle drawn from a normal distribution with a mean of zero and a standard deviation of 0.22 radians.

- Translation of a nucleotide, where the displacement along each Cartesian axis is drawn from a normal distribution with a mean of zero and a standard deviation of 0.15 simulation units of length (0.1277 nm).

For additional details relating to VMMC simulations of oxDNA, see also the supplemental material in references 7 and 11.

2. Thermodynamics: Umbrella Sampling

The free-energy profile as a function of an order parameter, Q, may provide useful information about a reaction or process, if an appropriate choice has been made. In particular, free-energy barriers can make certain regions of configuration space hard to reach, which prevents efficient sampling of all of the states of interest. The free-energy landscape can be artificially flattened by weighting states with different values of Q appropriately, a technique known as umbrella sampling.[12] Thermodynamic properties of the system can then be extracted from simulations by unweighting the resulting distributions.

In particular, for an unweighted simulation a particular microstate with coordinates $\mathbf{q}^N$ and energy $E(\mathbf{q}^N)$ is sampled with probability

$$P(\mathbf{q}^N) \propto e^{-\beta E(\mathbf{q}^N)}. \tag{S1}$$

The equilibrium average of some variable $A(\mathbf{q}^N)$ is then given by the sum over all states, weighted by their Boltzmann factors:

$$\langle A \rangle = \frac{\int A(\mathbf{q}^N) e^{-\beta E(\mathbf{q}^N)} d\mathbf{q}^N}{\int e^{-\beta E(\mathbf{q}^N)} d\mathbf{q}^N}. \tag{S2}$$

By applying a weighting $w = w(Q(\mathbf{q}^N))$ to each value of the order parameter, we change the sampling frequency to

$$P_w(\mathbf{q}^N) \propto w(Q(\mathbf{q}^N)) e^{-\beta E(\mathbf{q}^N)}. \tag{S3}$$

where the subscript w indicates a property of the weighted system. So we can artificially ensure that our simulation samples all states equally by making P_w constant for all microstates. Equilibrium thermodynamic properties are then obtained by unbiasing afterwards, as can be seen by rewriting Eq. (S2) as follows:

$$\langle A \rangle = \frac{\int \frac{A(\mathbf{q}^N)}{w(Q(\mathbf{q}^N))} w(Q(\mathbf{q}^N)) e^{-\beta E(\mathbf{q}^N)} d\mathbf{q}^N}{\int \frac{1}{w(Q(\mathbf{q}^N))} w(Q(\mathbf{q}^N)) e^{-\beta E(\mathbf{q}^N)} d\mathbf{q}^N} \tag{S4}$$

$$= \frac{\langle A/w \rangle_w}{\langle 1/w \rangle_w}. \tag{S5}$$

Additional details for augmenting umbrella sampling with VMMC simulations of oxDNA are also included in the supplemental material in references 7 and 11. Here, we refer to VMMC simulations augmented with umbrella sampling as VMMC-US simulations.

3. Molecular Dynamics

Dynamics simulations were performed using an Anderson-like thermostat, similar to the one described in Appendix A of reference 13. The Newtonian equations of motion for the system are integrated using a Verlet algorithm[14] with a discrete time-step δt, so that the positions, velocities, orientations, and angular velocities of the nucleotides are recalculated at each time-step. To achieve Brownian motion, the velocity of each nucleotide is resampled with a probability $p_v = 0.02$ from a Maxwell-Boltzmann distribution at the temperature of the solvent every 103 time steps. The algorithm also resamples angular velocities with a different probability $p_\omega = 0.0067$. On time scales longer than $N\delta t/p_v$, where δt is the integration time step and N is taken to be 103 steps, the dynamics is diffusive. We choose $\delta t = 1.52 \times 10^{-14}$s for all dynamics simulations in this study. For more details relating to molecular dynamics simulations of oxDNA, see the supplemental material in references 7 and 11. Here, we refer to simulations run with molecular dynamics as MD simulations.

4. Modeling the Origami Track in VMMC Simulations

All VMMC-US simulations were performed in a periodic box with $V = L^3$, where L was set to correspond to a concentration of $c_0 = 2.7\mu$M for the unbound F1 fuel strand, that is $V = 0.62\,\mu\text{L}^3$. Due to the large size of the origami, simulating the complete motor system using VMMC augmented with umbrella sampling is far too computationally challenging to achieve good sampling of the duplex-forming and duplex-dissociation events that are key to the operation of the motor. In order to efficiently sample the correct operation of the motor (raising/placing a walker foot onto the track), and the trapped-state scenario, we chose to use a simplified representation of the origami while continuing to use oxDNA to represent the walker, footholds and fuels. To achieve a realistic description, we first model the attachment of the footholds to a surface using a spring potential. We take the foothold strands in the simulations to be those bases that are complementary to the fuels plus the 3-base section that separates the complementary section from the origami (these bases are underlined in black in Table S1). The spring potential acts on the center-of-mass of an extra base (A) that we attached to the first $3'$ base of the foothold T1, and on the center-of-mass of an extra base (T) that we attached to the last $5'$ base of the foothold T2. The potential takes the form

$$V_{\text{spring}}(k, \mathbf{r}_1, \mathbf{r}_2) = \frac{k}{2}|\mathbf{r}_1 - \mathbf{r}_1^0|^2 + \frac{k}{2}|\mathbf{r}_2 - \mathbf{r}_2^0|^2, \tag{S6}$$

where $\mathbf{r}_1$ and $\mathbf{r}_2$ are the positions of the center of mass of extra base added to the first and last $3'$ and $5'$ nucleotide of the footholds T1 and T2, respectively, and $\mathbf{r}_1^0$ and $\mathbf{r}_2^0$ are the positions to which they are attached by the springs. We add this potential energy term to the total potential energy of the system. We model the different foothold positions by defining a set of coordinates where the attachment points

for T1 and T2 lie in the $z = 0$ plane, and are separated by a distance d in the x direction. In all simulations we chose a spring constant $k = 131\,\mathrm{pN\,nm^{-1}}$ which we have found in previous simulations of walkers to be a physically sensible choice that in this study should capture the behavior of the real system where the foothold strand is actually connected to a staple strand in the origami.[15]

We next mimic the presence of origami track by forbidding the walker, the footholds, and any fuels in the simulations from crossing the $z = 0$ plane. This is achieved by introducing a potential term that acts on the centers of mass of all nucleotides in a simulation, and takes the form

$$V^i_{\mathrm{repulsion}}(\mathbf{r}_i) = \begin{cases} \frac{k_r}{2} z_i^2, & \text{if } z_i < 0 \\ 0 & \text{if } z_i \geq 0 \end{cases} \tag{S7}$$

which we then add to the overall total potential of the system. We take $k_r = 1142\,\mathrm{pN\,nm^{-1}}$ which is large enough to prevent any nucleotide from crossing the $z = 0$ plane. In Fig. S2 we show several configurations of the walker having both feet placed onto our model of the track.

The binding of a second fuel to create a trapped state is a dimerization reaction, rather than a mono-molecular rearrangement such as the intended stepping reaction. The sampling of such transitions in the dilute limit is considered in detail in reference 16. If one of the reactants, A, is in an overwhelming excess relative to the other reactant B ($[A] >> [B]$), then it is possible to sample the behavior of a given B molecule by simulating a single pair of A and B in a box with a volume V chosen such that $1/V = [A]$. Moreover, it is even possible to extrapolate to other concentrations $[A]'$ by re-weighting the samples obtained so that unbound states are made relatively more likely by a factor $[A]/[A']$. In our case, the fuel is far in excess of the walker, and we use the above approach to infer free-energy profiles of fuel binding to the walker for a range of concentrations, all obtained from a single simulation in a volume $V = 0.62\,\mathrm{\mu L^3}$.

Our simulations have the additional complication that the walker is tethered to a plane at $z = 0$, with a repulsion for all nucleotides for $z < 0$. For simplicity, we apply repulsion to the absolute nucleotide coordinates, not the coordinates of a periodic image. Most of the strands are part of a tethered complex with absolute coordinates fixed within the first periodic cell, meaning that this decision has no effect. However the fuel strand F1 can diffuse freely in trapping simulations, and hence can possess absolute coordinates that imply $z < 0$ when translated back to the first periodic cell. The result is that the free strand really does sample the whole volume V, which is important when applying the concentration scaling discussed above.

One might be concerned that the above approach allows F1 to bind to the walker while violating the repulsive plane. In practice, however, such configurations are rare since the complementary strand would also tend to have coordinates with $x < 0$, and this is still penalized. To confirm this we checked the configurations in trajectories from VMMC-US simulations that had 1 base pair intact between L1 and F1 strands, and found that less than 1% of them had some section of the F1 strand violating the plane.

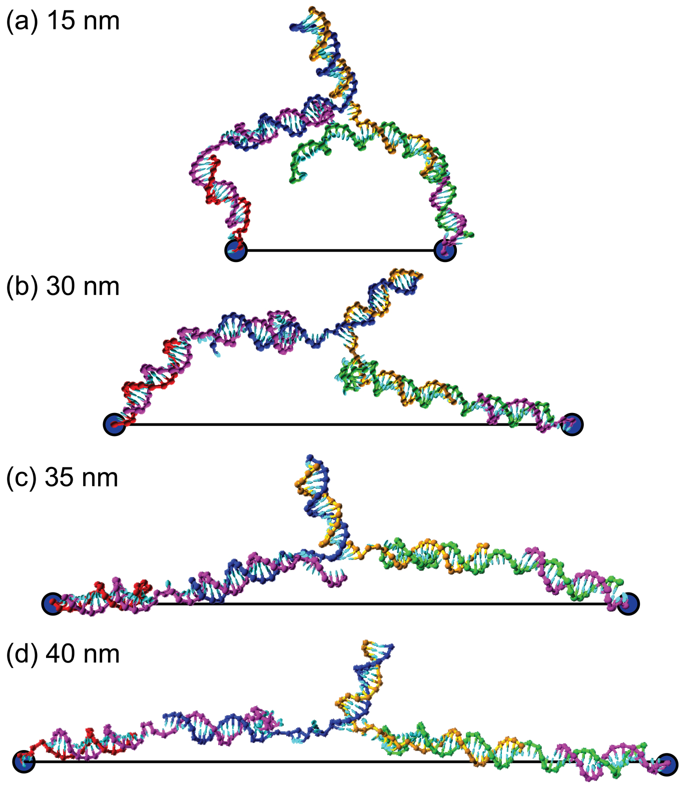

FIG. S2. Example configurations of the walker when both feet are placed onto the track for step sizes (a) 15 nm, (b) 30 nm, (c) 35 nm, and (d) 40 nm. Even though it may appear that DNA may pass below the $z = 0$ plane in (b) and (d), this is an artifact of our choice of the viewing angle which we selected to best illustrate the configuration.

5. Simulations of the Origami on a Surface

We investigated the origami when it was absorbed onto a surface in MD simulations, as is the case when visualized by AFM and other types of microscopy. This has the effect of rendering the origami mostly flat as the configuration in Fig. S3 illustrates. The interaction of the nucleotides with the surface is modeled with a simple 1D Lennard-Jones interaction that depends only on the distance of a nucleotide from the surface (in our simulations the surface is the xy-plane at $z = 0$). The potential has the form

$$V^i_{\mathrm{LJ}}(\mathbf{r}_i) = 4\epsilon \left[\left(\frac{\sigma}{z_i}\right)^{2n} - \left(\frac{\sigma}{z_i}\right)^{n} \right] \tag{S8}$$

where n is an integer, σ is the inter-particle distance when the potential is zero, and ϵ is the well depth. We chose to use $n = 2$, $\sigma = 1$, and $\epsilon = 2.85\,\mathrm{pN\,nm^{-1}}$. We only use Eq. S8 as a simple way to constrain the nucleotides to a plane; it is not meant to provide a realistic representation of a particular surface. We also constrained the origami in between two repulsive planes using Eq. S7 and found similar results when compared with the Lennard-Jones surface potential, which suggests that the results do not depend strongly on the particular form of the potential used.

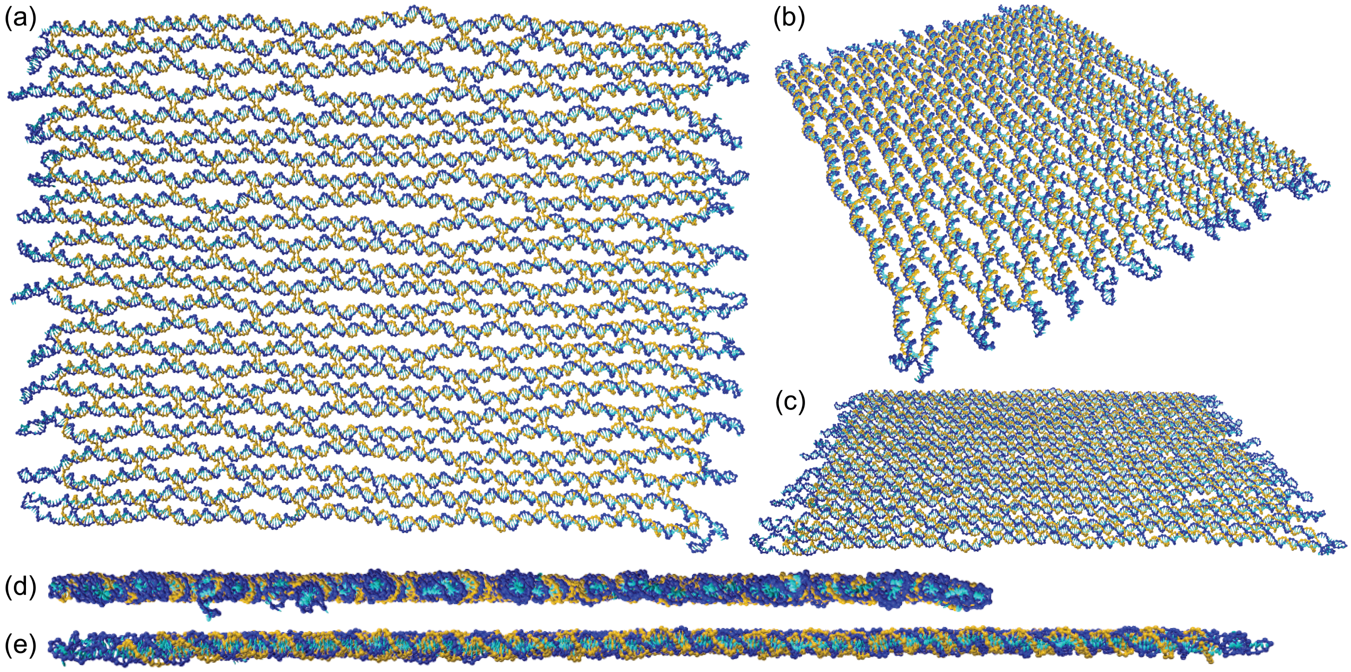

FIG. S3. Example of an oxDNA configuration of the origami track when placed onto a surface. (a-c) Viewpoints showing the top of the origami, (d,e) side views of the origami looking at the helices either (d) end-on or (e) side-on.

C. Simulation Protocols

In this section we discuss the implementation of the algorithms of Section SII B for the motor system. In our simulations we employed a 'distance order parameter' that measures the minimum distance between hydrogen-bonding sites over any inter-strand pairs of complementary bases between the walker and a fuel strand. We also used a 'bonds' order parameter that is capable of measuring the total number of intra-strand base pairs for strands, and inter-strand base pairs between two strands. The definition of a bonded base pair in our simulations is two bases with a hydrogen bonding energy below 0.596 kcal/mol. This value for the selected cutoff corresponds to about 15% of the typical hydrogen-bond energy for a base pair. The different order parameters listed above are combined into multi-dimensional quantities for use in a simulation.

1. Thermodynamics

In order to characterize the influence of step size and fuel concentration on motor performance, we ran simulations for two scenarios. The first scenario (call it A) captures the correct operation of the motor. We sample the walker, which has one foot already bound to the track at T2, placing its other foot down at T1. We also sample the walker raising its foot at T1 given that both feet are initially placed onto the track. In the second scenario (call it B), which involves the trapped state, we first assume that an F1 fuel is bound to T1. We then sample the attachment of a second F1 fuel to the walker, as well as the dissociation of an F1 fuel from the walker.

The simulations for the two scenarios were carried out using VMMC moves along with umbrella sampling to encourage the formation and breaking of base pairs. A multi-dimensional order parameter was used which tracks the minimum distance between the complementary bases between the walker and the fuel (L1F1 base pairs), and the total number of base pairs between between L1 and F1. Using the simulation results, we compute the free-energy difference between the bound and unbound states involving L1 and F1, $\Delta G^0 \equiv G_{\geq \text{ 1 b.p.}} - G_{0 \text{ b.p.}}$, and the activation barrier, $\Delta G^\ddagger \equiv G_{1 \text{ b.p.}} - G_{0 \text{ b.p.}}$, for both scenarios. These quantities are used to compute the yield of the walker in its foot-down state as a function of step size, which is discussed below in Section SII C 2.

In order to sample the formation and breaking of base pairing between L1 and F1 more efficiently, we split simulations of scenario A into two windows that separately sample: (1) the leg-lifted state, where F1T1 is intact and L1 has formed zero or one base pair(s) with F1; and (2) the leg-placed state, where at least one base pair has formed between L1 and F1 and F1T1 is intact. Similarly, in scenario B we sampled: (1) the leg-lifted state, where the fuel F1 is free in solution at a concentration of 2.7μM, and the state where L1 and F1 have one base pair between them, where in both bound and unbound states it is assumed that F1 is blocked from binding with T1; (2) the state where F1 is bound to L1 but not bound to T1, where L1 and F1 have formed at least one base pair. When simulations for the two windows in either scenario have converged, they can be recombined by using the weighted histogram method (WHAM).[17]

During preliminary simulations, we also observed the formation of intra-strand base pairs in the free single-stranded

section of the F1 fuel, and in the single-stranded section of the walker (L1) when in the foot-up state. To capture any effects that the secondary structure might have on motor performance, we ran simulations for all of the scenarios described in the previous paragraph for two cases: (1) any complementary base pair is allowed to form, which includes intra-strand base pairs, the designed inter-strand base pairs between L1 and F1, and other inter-strand base pairs (misbonds) between these two strands; (2) only the designed base pairs between L1 and F1 are allowed to form, thus preventing possible secondary structure and misbonding occurring in the simulations. To simulate the first case, we modified the multi-dimensional order parameter discussed above to include an extra dimension that tracked the number of intra-strand base pairs in the F1 fuel. This allowed us to explicitly bias the formation and breaking of these base pairs during VMMC simulations augmented with US. We also tracked the number of intra-strand base pairs in the single-stranded L1 section of the walker when in its foot-up state. However, we observed this secondary structure to be relatively transient during preliminary simulations, so we chose not to explicitly encourage the formation/breaking of these base pairs like we did for F1 (but secondary structure was not prevented from forming or breaking). The results of the VMMC simulations combined with US are discussed in Section SII D.

For all of the different scenarios and their respective windows described above, we ran multiple identical simulations for at least 2.33×10^{10} steps. The results from these simulations are shown in Figs. 2 and 3 in the main text, and in Figs. S4-S7 in Section SII D.

2. Calculation of the Yield

As noted, we use the results from the simulations described above to compute the yield of the walker in its foot-down state. As the binding of the F1 strand is thermodynamically very favorable, the kinetic yield is the more appropriate quantity to compare with experiment. We computed the kinetic yield using the activation barrier energies for leg-placing ($\Delta G_s^\ddagger$) and multi-fuel binding reactions ($\Delta G_{FB}^\ddagger$). These activation energies are related to the rates for leg-placing (k_s) and multi-fuel-binding (k_{FB}) reactions through

$$k_s = k_{s,0} \exp\left(-\Delta G_s^\ddagger / k_{\mathrm{B}}T\right) \tag{S9}$$

$$k_{FB}(c) = k_{FB,0} \exp\left(-\Delta G_{FB}^\ddagger(c) / k_{\mathrm{B}}T\right) \tag{S10}$$

where $k_{s,0}$ and $k_{FB,0}$ are constants, respectively. The activation barrier $\Delta G_{FB}^\ddagger$, and hence k_{FB}, explicitly depends on the concentration, c, of the fuel.

The kinetic yield measured in the experiments (Eq. 1 in the main text) is

$$Y_{\mathrm{kin}} = \frac{k_s}{k_s + k_{FB}(c)} \tag{S11}$$

$$= \frac{k_{s,0} e^{-\Delta G_s^\ddagger / k_{\mathrm{B}}T}}{k_{s,0} e^{-\Delta G_s^\ddagger / k_{\mathrm{B}}T} + k_{FB,0} e^{-\Delta G_{FB}^\ddagger(c) / k_{\mathrm{B}}T}}. \tag{S12}$$

To simplify Eq. S12, we note that the relative value of the rates, $k_s/k_{FB}(c)$, and hence the kinetic yield, is primarily determined by the difference in the activation energies $\Delta G_s^\ddagger - \Delta G_{FB}^\ddagger$, and not by the value of the ratio $k_{s,0}/k_{FB,0}$. Furthermore, as we are comparing similar processes with similar microscopic kinetics, it is reasonable to assume $k_{s,0} \approx k_{FB,0}$. With this approximation, Eq. S12 simplifies to

$$Y_{\mathrm{oxDNA}} \approx \frac{e^{-\Delta G_s^\ddagger / k_{\mathrm{B}}T}}{e^{-\Delta G_s^\ddagger / k_{\mathrm{B}}T} + e^{-\Delta G_{FB}^\ddagger(c) / k_{\mathrm{B}}T}} \tag{S13}$$

which we use to compare with experimental measurements. Note that Eq. S13 only depends on the difference in the free-energy barriers, and not their absolute values. In particular, we expect the differences in free-energy barriers in our simplified reaction coordinate system are a reasonable reflection of differences in free energy of transition ensembles for distinct but related systems.[18] Following a similar argument, we compute the stepping rate at different step sizes relative to the 10 nm step size as $k_s(d)/k_s(10\,\mathrm{nm}) = \exp(-(\Delta G_s^\ddagger(d) - \Delta G_s^\ddagger(10\,\mathrm{nm}))/k_{\mathrm{B}}T)$.

In addition to the kinetic yield we also computed the corresponding equilibrium yield, Y_{oxDNA}^0, by utilizing the free-energy differences ΔG_s^0 and ΔG_{FB}^0 measured in the simulations. The equilibrium yield is given by

$$Y_{\mathrm{oxDNA}}^0 = \frac{e^{-\Delta G_s^0 / k_{\mathrm{B}}T}}{1 + e^{-\Delta G_s^0 / k_{\mathrm{B}}T} + e^{-\Delta G_{FB}^0(c) / k_{\mathrm{B}}T}} \tag{S14}$$

where we have made the concentration dependence of ΔG_{FB}^0 explicit. We note that both kinetic and equilibrium yields show very similar behavior (see Fig. S4). This is because the variation in the activation free energy is the main cause of the differences in the overall free-energy changes.

Both Eqs. S13 and S14 require the relevant free energies to be computed as a function of the fuel concentration. As noted previously, in the computation of both Eq. S13 and S14, we simulated the multi-fuel binding scenario at a fixed concentration of $c_0 = 2.7\mu$M. Since the ratio of bound to unbound states is proportional to c_0, the results can be extrapolated to another concentration, c_i, by rescaling the bound-to-unbound ratio by a factor of (c_i/c_0). The activation barrier or equilibrium free-energy is then computed at the new concentration. The quantity Y_{oxDNA}^0 is shown in Fig. 3 in the main text for the concentrations of F1 used in the experiments and when secondary structure and misbonds are allowed to form in simulations. Additionally, in Fig. S4 we show the kinetic and equilibrium yields for the case when secondary structure and misbonds are allowed to form in simulations, and the case when only the designed L1F1 base pairs are allowed to form.

3. Calculation of Average Step Sizes

To provide a measure of the step size of the walker for the different possible foothold positions we computed the distance between the attachment points of the relevant footholds

during long MD simulations that had to be run on GPUs due to the large size of the origami. Specifically, we used for a foothold location on the origami the center-of-mass of the base that is contained within the origami but which is connected to the single-stranded three-base section of a foothold. The dangling footholds themselves are not simulated. The step size is then taken to be the distance measured between the center-of-mass of the base at the location of T1 (green dot in Fig. 1 in the main text) and the center-of-mass of the base at the location of another foothold (red dots in Fig. 1). During the simulations we periodically measured each designed step size (from 5 nm to 40 nm, see Fig. 1 in the main text) for a given configuration, constructed histograms and computed average values for both long and short axes.

The distributions $p(d)$ for each step size d are shown in Fig. 5 in the main text for the origami. In Fig. S8 we show comparable quantities when the origami is absorbed onto a 'surface' in the simulations, rendering it roughly flat. We measured the distribution for each step size by running long simulations, and then computed the mean and standard deviation of the distribution. In the second column of Table SII, we list the mean and standard deviation for all step sizes measured. In Table SIII we list the mean and standard deviation for computed distances between each pair of neighboring footholds. Additionally, in the third column of Table SII we list the mean and standard deviation for the step sizes for the origami that is attached to a surface. The distributions for the foothold separations when the origami is on the surface are very similar to the free origami for the long axis, but show much narrower distributions for the short axis due to the suppression of bending fluctuations.

4. Calculation of Walker and Foothold Ranges

We used long, unbiased VMMC simulations to determine the extent to which the walker with one foot bound to the track and the other free (denoted L1F2T2), and the F1T1 foothold-bound fuel, are able to sample their local environments. For the F1T1 complex we define the 'range distance' d_1 to be the distance between the center-of-mass of the terminal base of foothold T1 that is attached to the track via a spring force, and the center-of-mass of the terminal base in the F1 strand that is attached to T1. The F1T1 'range' R_1 is defined to be the distance for which 95% of configurations had $d_1 \leq R_1$. Similarly, for the L1F2T2 complex we define the 'range distance' d_2 to be the distance measured between the center-of-mass of the base in foothold T2 that is attached to the track via a spring force, and the center-of-mass of the terminal base in the walker strand L1, given that F2 was fully bound to T2. The L1F2T2 'range' R_2 is defined to be the distance for which 95% of configurations sampled in simulations had $d_2 \leq R_2$.

During the simulations we periodically measured d_1 and d_2 for a given configuration, and then computed the mean and standard deviation for d_1, d_2, R_1, and R_2 for the case when secondary structure and misbonds were allowed to form in simulations, and the case when only the designed base pairs were allowed to form. These quantities are listed in Table SIV. In Fig. 4 in the main text, the gray circles illustrate R_1 and R_2 for the case when secondary structure was not sampled in the simulations. Finally, histograms of d_1 and d_2 are shown in Fig. S9.

D. Supplemental Results

1. Computed Free-energy Landscapes and Yields

In Fig. S4 we plot the kinetic and equilibrium yields computed using Eq. S13 (left plots) and Eq. S14 (right plots), respectively, for the cases when only the designed F1T1 base pairs were allowed to form (top plots), and when secondary structure and misbonds were allowed to form (bottom plots).

In Fig. S5(a) and (b) we plot free-energy landscapes for step sizes for the cases when only the designed F1T1 base pairs were allowed to form, and when secondary structure and misbonds were allowed to form, respectively. In Fig. S6 we plot the activation barrier $\Delta G^{\ddagger}$ versus step size for the case when only the designed F1T1 base pairs were allowed to form. In Figs. S7(a) and (b) we plot ΔG^{0} versus step size when only the designed F1T1 base pairs were allowed to form, and when secondary structure and misbonds were allowed to form, respectively.

Comparing Figs. S4(a) and (b), and Figs. S4(c) and (d) shows that the computed yield is not very sensitive to the presence of secondary structure. However, the plots also show that the yield is slightly lower when computed using $\Delta G^{\ddagger}$ rather than ΔG^{0}. This is true regardless of whether or not secondary structure was allowed to form in simulations, but is clearly more noticeable when it is present (compare Figs. S4(a) with (c) and Figs. S4(b) with (d)).

2. Range Calculation Results

Table SIV lists the mean and standard deviation for the distributions of d_1 and d_2, and the ranges R_1 and R_2. Fig. S9 shows the histograms for the probability densities $\rho(d_1) \equiv p(d_1)/d_1^2$ and $\rho(d_2) \equiv p(d_2)/d_2^2$ for the case when only the designed F1T1 base pairs were allowed to form (top plot), and when secondary structure and misbonds were allowed to form (bottom plot). Fig. S10 illustrates typical configurations of L1F1 when secondary structure is present in the F1 strand.

The top plot in Fig. S9 clearly shows that both L1F1 and L1F2T2 sample a large range with broad overlap between the distributions, while the bottom plot shows that when secondary structure is present the distribution for F1T1 is restricted to a much smaller range, with almost no configurations having distance values less than 5 nm or greater than 15 nm. There is considerably less overlap between the two distributions, though this is mostly caused by changes to $\rho(d_1)$ due to the secondary structure, as Table SIV shows that there is not much difference in the average values of d_1 or R_1 for the two scenarios, and the densities $\rho(d_2)$ are also similar in shape.

Fig. S10 illustrates configurations in (i-ii) having $d_1 < \langle d_1 \rangle$, (iii-v) having $d_1 \approx \langle d_1 \rangle$, and (vi-vii) having $d_1 > \langle d_1 \rangle$. All

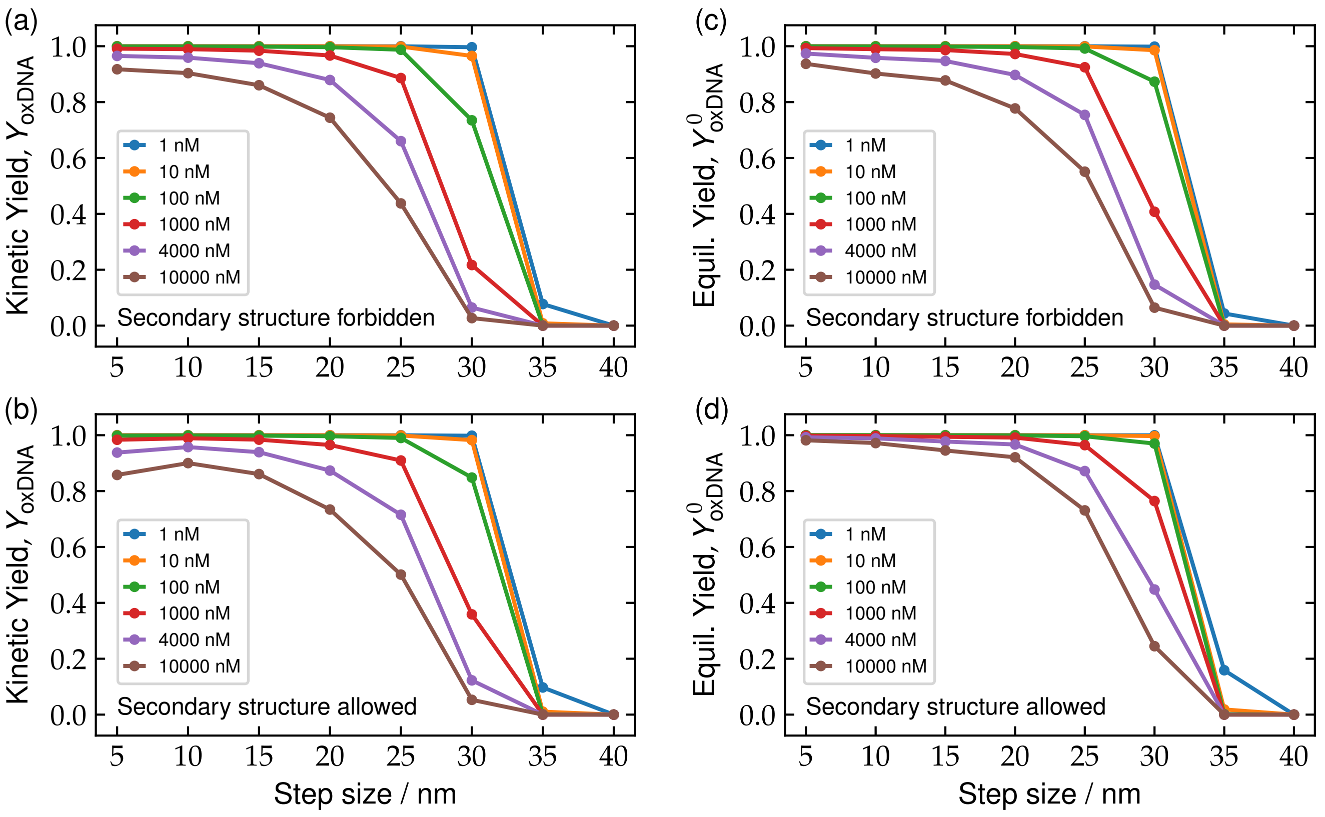

FIG. S4. The kinetic yield versus step size is shown for cases when: (a) only the designed F1T1 base pairs were allowed to form; (b) secondary structure and misbonds were allowed to form. The equilibrium yield versus step size is shown for cases when: (c) only the designed F1T1 base pairs were allowed to form; (d) secondary structure and misbonds were allowed to form.

Long Axis		
Designed	Free Origami	Flat Origami
5	6.07 ± 0.31	6.13 ± 0.28
10	11.04 ± 0.20	11.14 ± 0.16
15	16.19 ± 0.28	16.40 ± 0.22
20	21.66 ± 0.26	21.90 ± 0.20
25	26.61 ± 0.34	27.06 ± 0.23
30	32.18 ± 0.35	32.70 ± 0.22
35	36.67 ± 0.44	37.50 ± 0.23
40	42.94 ± 0.44	43.85 ± 0.23
Short Axis		
Designed	Free Origami	Flat Origami
5	5.69 ± 0.48	5.58 ± 0.42
10	11.21 ± 0.66	11.20 ± 0.50
15	16.42 ± 0.91	16.85 ± 0.56
20	21.11 ± 1.36	22.45 ± 0.61
25	25.44 ± 1.94	28.10 ± 0.65
30	29.10 ± 2.68	33.75 ± 0.70
35	32.15 ± 3.57	39.39 ± 0.75
40	34.55 ± 4.63	45.01 ± 0.80

TABLE SII. Left: Designed step sizes. Middle: Computed step sizes for the origami track. Right: Computed step sizes for the origami track held to a surface in simulations. All measurements are in nm. Note that the quantities that involve ± refer to the mean and standard deviation of the measured distribution.

Short Axis	
Neighboring Footholds	Mean Step Size
0-5	5.69 ± 0.48
5-10	5.99 ± 0.59
10-15	5.89 ± 0.60
15-20	6.07 ± 0.58
20-25	5.86 ± 0.59
25-30	6.01 ± 0.58
30-35	5.92 ± 0.59
35-40	6.01 ± 0.63

TABLE SIII. Left: Designed foothold pairs along the short axis of the origami. Right: Computed step sizes for the foothold pairs. The mean step size for a foothold pair is 5.91 ± 0.16 nm. All measurements are in nm. Note that the quantities that involve ± refer to the mean and standard deviation of the measured distribution.

configurations illustrate the presence of secondary structure, ranging from 4 to 7 intra-strand base pairs. In (ii-v) the configurations show that nearly all of the bases in the L1 section are sequestered, but only 6 or 7 base pairs are typically present. These states are also the most typical at equilibrium, having occupancy probabilities of 30% and 29%, respectively. The remaining configurations show 4 or 5 inter-strand base pairs mainly between the bases in the middle section of the

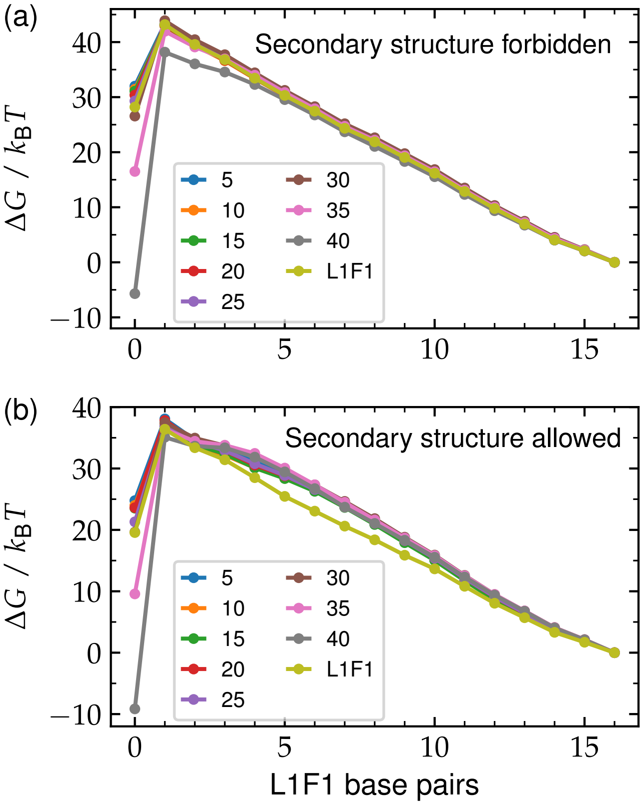

FIG. S5. Free-energy landscapes versus the total number of base pairs between L1 and F1 strands for the cases when: (a) only the designed F1T1 base pairs were allowed to form; (b) secondary structure and misbonds were allowed to form. In both plots the concentration of L1F1 was 100 nM. The label L1F1 in the legend refers to the landscape for the scenario when F1 binds to L1 but is not otherwise bound to a foothold. The remaining legend labels refer to the step size.

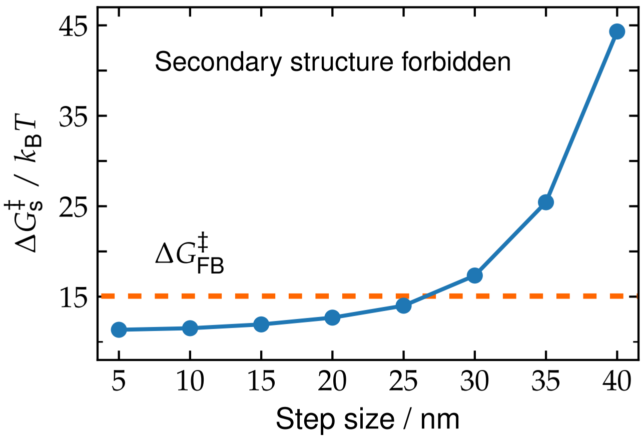

FIG. S6. Activation barrier $\Delta G^{\ddagger}$ vs step size for the case when only the designed F1T1 base pairs were allowed to form. The dashed (orange) horizontal line denotes the value of $\Delta G^{\ddagger}$ for L1F1 at 100 nM.

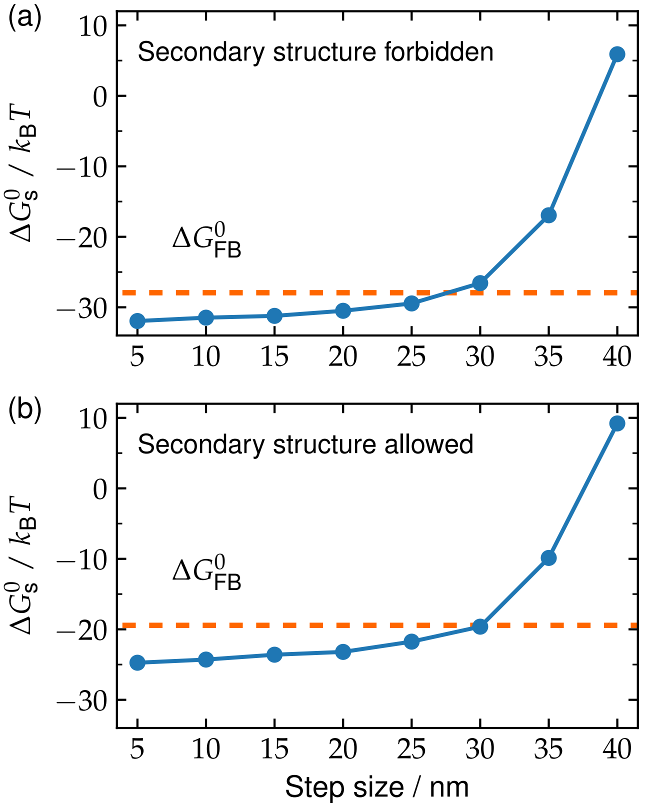

FIG. S7. ΔG^0 versus step size for cases when: (a) only the designed F1T1 base pairs were allowed to form; (b) secondary structure and misbonds were allowed to form. In both plots, the dashed (orange) horizontal line denotes the value of ΔG^0 for L1F1 at 100 nM.

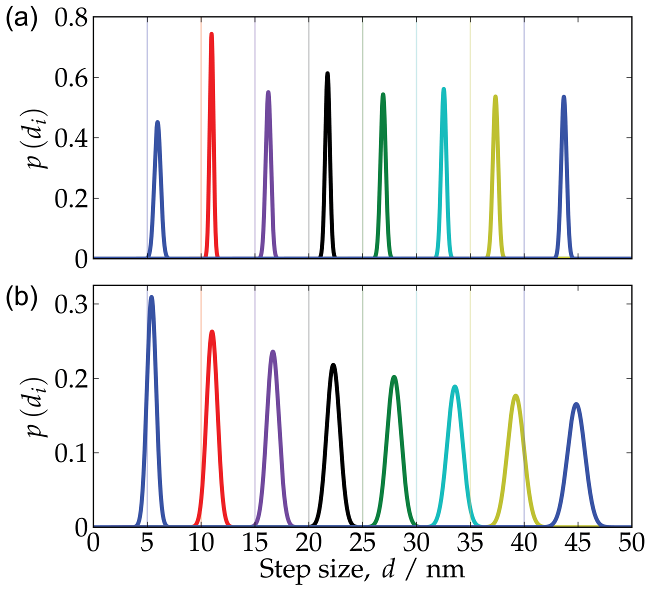

FIG. S8. Histograms for the step sizes when the origami has been placed onto a surface in the simulations. Results are shown for (a) the long axis and (b) the short axis. Vertical lines denote the designed foothold positions.

Quantity	Allowed	Forbidden
$\langle d_1 \rangle$	15.99 ± 3.01	15.29 ± 3.79
$\langle d_2 \rangle$	8.51 ± 1.77	11.53 ± 3.04
R_1	20.63 ± 0.69	21.03 ± 0.10
R_2	11.75 ± 0.95	16.16 ± 0.01

TABLE SIV. Mean and standard deviation are reported for the distributions of d_1 d_2, R_1 and R_2 for simulations when secondary structure and misbonds were allowed to form, and when only the designed base pairs F1T1 base pairs were allowed to form (i.e. secondary structure was forbidden from forming). These scenarios are labeled 'Allowed' and 'Forbidden', respectively. The subscripts 1 and 2 refer to F1T1 (foothold-bound fuel) and L1F2T2 (walker), respectively. All reported calculations are in nm. Note that quantities that involve ± refer to the mean and standard deviation of the measured distribution.

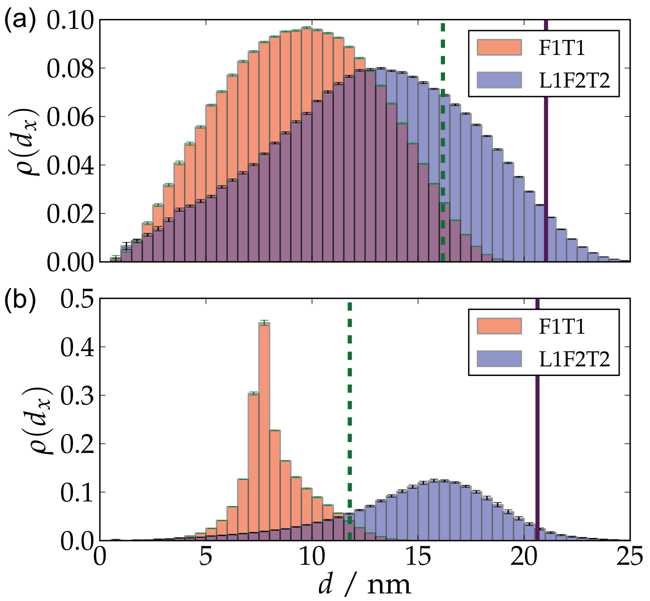

FIG. S9. Histograms of $\rho(d)$ for the walker (L1F2T2) and the foothold-bound fuel (F1T1) are shown for cases when: (a) only the designed base pairs F1T1 base pairs were allowed to form; (b) secondary structure and misbonds were allowed to form. In both figures we use the label $x = 1, 2$ to refer to the range distances d_1 and d_2 for F1T1 and L1F2T2, respectively. Additionally, dashed (green) lines denote the value of R_1 while solid (purple) lines denote the value of R_2.

L1 strand. These states have occupancy probabilities of 19% and 14%, respectively. The state with no secondary structure has an occupancy probability of only ∼1%.

Secondary structure stabilizes the unbound states, leading to larger magnitudes for Delta G when secondary structure is forbidden. However, as these changes affect both the leg-placing and fuel binding rates similarly, the equilibrium yields are very similar in the two cases. In the free-energy profiles the secondary structure leads to noticeable non-linearities that are associated with the loss of secondary structure if the double helices are to further zip up.[7]

3. CanDo Comparisons

As the effective pitch of the Rothemund tile is 10.67 base pairs per turn, which is greater than the natural DNA pitch of 10.5 base pairs per turn, the tiles are expected to be twisted. For an approximately 2-dimensional object like the current DNA origami it is very likely that this twist is coupled to bend. There are two basic ways that the origami can bend having different signs for the curvature. For objects in which there is no difference between front and back faces, the two bending modes are equivalent. However for a DNA origami the two faces are different, and so therefore the two modes of bending are distinct.

We compared the preferential bending direction of the origami as predicted by oxDNA with the predictions of CanDo.[19] Fig. S11 shows that oxDNA's predicted bending direction, namely with the walker being on the convex side of the origami, is consistent with the CanDo predicted structure (the figure shows a helix positioned on the same side as the walker). Both CanDo and oxDNA are also consistent with the inferred bending for the specific tiles studied in references 20 and 21. We also varied the bending and torsional stiffnesses in CanDo to probe for the cause of the directional bending, but observed the same preference even when setting the stiffness parameter to a value that makes the nicks mechanically equivalent to the rest of the duplex. This suggests that the nicks, which are all on the same side of the origami, are probably not the cause of the bending. The bending preference must be a result of the detailed mechanical properties of the DNA double helices and their couplings through the junctions, but we were not able to provide a simple rationalization of the preference.

4. Movies

In the web-enhanced content we provide one movie illustrating the walker in the foot-up state sampling its local environment, and one movie showing the foot-up walker placing its free leg onto the origami track by forming base pairs between the F1 and T1 strands. In both movies the step size is 15 nm. Each movie was generated by running dynamics simulations while periodically saving configurations.

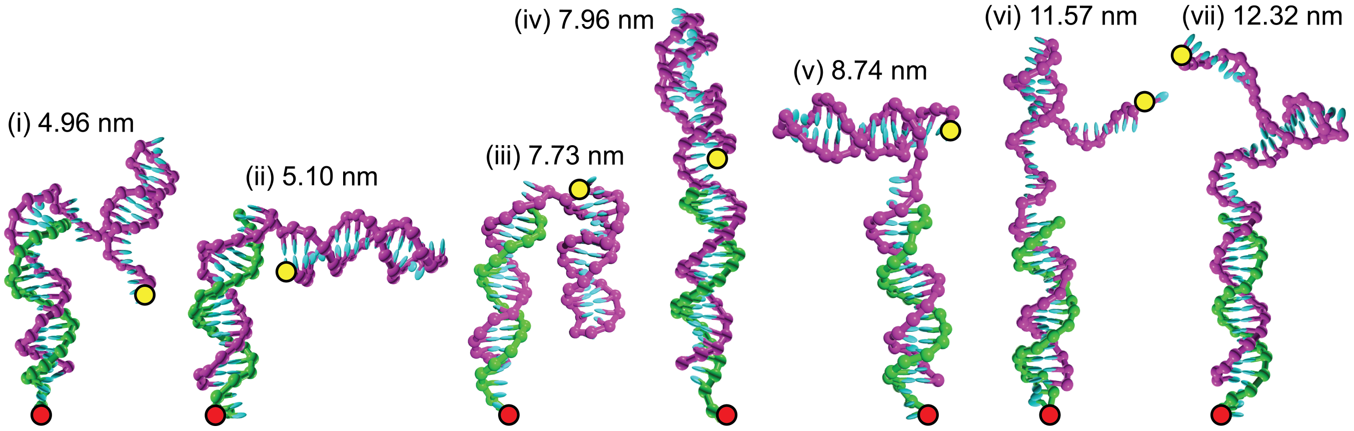

FIG. S10. Configurations of the F1T1 complex showing hairpin structure in the F1 strand (purple). The reported values are the measured end-to-end distance (measured from the red dot to the yellow dot) of the F1T1 complex.

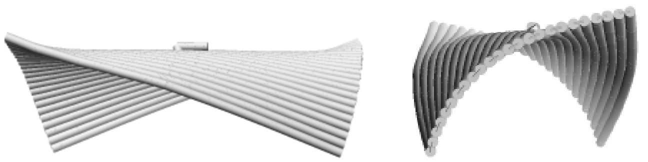

FIG. S11. Two views of the CanDo predictions of the structure of the origami tile considered here. An extra helix has been added to the face of the origami on which the walker is attached to help differentiate the two sides. Like oxDNA, CanDo predicts that the walker will be on the convex face of the origami, although the degree of bending is less than seen with oxDNA.

[1] S. F. J. Wickham, J. Bath, Y. Katsuda, M. Endo, K. Hidaka, H. Sugiyama, and A. J. Turberfield, Nat. Nanotechnol. **7**, 169 (2012).
[2] T. E. Tomov, R. Tsukanov, M. Liber, R. Masoud, N. Plavner, and E. Nir, J. Am. Chem. Soc. **135**, 11935 (2013).
[3] M. Liber, T. E. Tomov, R. Tsukanov, Y. Berger, and E. Nir, Small **11**, 568 (2015).
[4] P. W. K. Rothemund, Nature **440**, 297 (2006).
[5] T. E. Tomov, R. Tsukanov, R. Masoud, M. Liber, N. Plavner, and E. Nir, Biophys. J. **102**, 1163 (2012).
[6] B. E. K. Snodin, F. Randisi, M. Mosayebi, P. Šulc, J. S. Schreck, F. Romano, T. E. Ouldridge, R. Tsukanov, E. Nir, A. A. Louis, and J. P. K. Doye, J. Chem. Phys. **142**, 234901 (2015).
[7] J. S. Schreck, T. E. Ouldridge, F. Romano, P. Šulc, L. P. Shaw, A. A. Louis, and J. P. K. Doye, Nucleic Acids Res. **43**, 6181 (2015).
[8] `https://dna.physics.ox.ac.uk`.
[9] P. Šulc, F. Romano, T. E. Ouldridge, L. Rovigatti, J. P. K. Doye, and A. A. Louis, J. Chem. Phys. **137**, 135101 (2012).
[10] S. Whitelam, E. H. Feng, M. F. Hagan, and P. L. Geissler, Soft Matter **5**, 1251 (2009).
[11] T. E. Ouldridge, P. Šulc, F. Romano, J. P. K. Doye, and A. A. Louis, Nucleic Acids Res. **41**, 8886 (2013).
[12] G. M. Torrie and J. P. Valleau, J. Comp. Phys. **23**, 187 (1977).
[13] J. Russo, P. Tartaglia, and F. Sciortino, J. Chem. Phys. **131**, 014504 (2009).
[14] L. Verlet, Phys. Rev. **159**, 98 (1967).
[15] P. Šulc, T. E. Ouldridge, F. Romano, J. P. Doye, and A. A. Louis, Nat. Comput. **13**, 535 (2014).
[16] T. E. Ouldridge, A. A. Louis, and J. P. Doye, J. Phys. Condens. Matter **22**, 104102 (2010).
[17] S. Kumar, J. M. Rosenberg, D. Bouzida, R. H. Swendsen, and P. A. Kollman, J. Comp. Chem. **13**, 1011 (1992).
[18] M. Mosayebi, A. A. Louis, J. P. K. Doye, and T. E. Ouldridge, ACS Nano **9**, 11993 (2015).
[19] D.-N. Kim, F. Kilchherr, H. Dietz, and M. Bathe, Nucl. Acids Res. **40**, 2862 (2011).
[20] A. Johnson-Buck, J. Nangreave, D.-N. Kim, M. Bathe, H. Yan, and N. G. Walter, Nano Lett. **13**, 728 (2013).
[21] Z. Li, L. Wang, H. Yan, and Y. Liu, Langmuir **28**, 1959 (2011).
